# Physical Processes in Polymeric Filters Used for Dialysis †

**DOI:** 10.3390/polym11030389

**Published:** 2019-02-26

**Authors:** Marina Voinova, Nikolay Repin, Evgen Sokol, Bogdan Tkachuk, Leonid Gorelik

**Affiliations:** 1 Department of Physics, Chalmers University of Technology, 41296 Gothenburg, Sweden; 2 Department of Industrial and Biomedical Electronics, Kharkiv Polytechnical Institute, National Technical University, 61002 Kharkov, Ukraine; sokol@kpi.kharkov.ua; 3 Department of Cryomorphology, Institute for Problems of Cryobiology and Cryomedicine, 61015 Kharkov, Ukraine; nvrepin@mail.ru; 4 Department of Hemodialysis, Municipal Noncommercial Enterprise of Kharkiv Regional Council “Regional Medical Clinical Center of Urology and Nephrology n.a. V.I. Shapoval”, 61037 Kharkov, Ukraine; bog.tkachuk@gmail.com

**Keywords:** polymeric membranes, capillary filters, physical analysis of transport, cell–polymeric surface interaction, shape of red blood cells, dialysis

## Abstract

The key physical processes in polymeric filters used for the blood purification include transport across the capillary wall and the interaction of blood cells with the polymer membrane surface. Theoretical modeling of membrane transport is an important tool which provides researchers with a quantification of the complex phenomena involved in dialysis. In the paper, we present a dense review of the most successful theoretical approaches to the description of transport across the polymeric membrane wall as well as the cell–polymer surface interaction, and refer to the corresponding experimental methods while studying these phenomena in dialyzing filters.

## 1. Introduction

Biological filters of the kidney are very complex. Artificial membranes that partially replace the renal function of the organism, being modeled after their natural prototypes, are also quite sophisticated. The present paper summarizes various theoretical approaches describing the transport of fluid and solutes in artificial membranes, bridging physics and biophysics in order to collect different parts of the puzzle and bring light to the complex mechanism of membrane transport. It is aimed at both specialists (dialysis researchers, doctors working in the field of dialysis and blood purification, students of biomedical and life sciences) as well as wider audience with interest in physical processes in biological and polymer membranes. The purpose of the current review is to introduce the generalized theoretical physics view to the quantitative analysis of molecular transport in such complex systems as natural and synthetic membrane filters in dialysis and physical processes involved in the cell-artificial surface interaction. The review is written to provide theoretical support for and facilitate the navigation of the interested readers in the modeling of membrane filtration in dialysis.

Section 2, which opens the current review, is devoted to the history of dialysis. Section 3 provides an introduction to the transport of water across natural filter membranes in the organism. In it, mechanisms of glomerular and peritoneal transport are compared.

Section 4 describes the mathematical modeling of the transport of water and solutes in both types of dialysis based on general thermodynamic principles of non-equilibrium thermodynamics. The classical two pore model and the distributed model are analyzed in details. In parallel, both the three pore model (TPM) of peritoneal membrane transport and the extended TPM theory with applications to the automated peritoneal dialysis are presented.

Section 5 provides an overview of physical processes in synthetic membrane filters used in hemodialysis. Mathematical modeling of molecular transport across polymeric dialysis membranes and diffusion across the tortuous membrane pathways in fouling layers is briefly reviewed in Section 6.

Cell adhesion and related shape transformation on artificial surfaces is a central question in the search for biocompatible polymers and, in particular of blood contacting materials used in dialysis. In Section 7, we present the basic physico-mathematical models of closed membranes shape originating from Helfrich’s curvature elasticity theory. In parallel, new developments in the red blood cell shape description are discussed. The calculated shapes are compared with the experimentally observed cell’s morphology on polymeric surfaces.

The last two sections contain a discussion of models not reviewed in the main text and a brief summary of the theoretical results.

## 2. Notes on the History of Dialysis

### 2.1. Hemodialysis

The first hemodialysis (HD) treatment of a human patient was conducted in Hessen in October 1924 by the German physician Georg Haas [1,2]. He used hirudin as the anticoagulant substance due to the fact that heparin was not yet accessible [3]. Haas’s first dialyzer of U-shaped collodion tubes was a construction immersed into a glass cylinder with a bath solution [2] during the first 15 min treatment time of the patient. In 1925 Haas wrote a short report describing the blood purification process performed on a human patient, the first hemodialysis attempt in the history of medicine [2]. Heparin became available in the 1930s [3] and Haas used this anticoagulant in his experiments instead of hirudin. The Collodion (or celloidin, cellulose-trinitrate) membrane method of preparation has been developed by Fritz Pregl, an Austrian chemist, Nobel Prize winner in 1923 [4]. Haas wrote:

“I have tried a series of different dialyzers from a variety of materials, animal and vegetable membranes and paper dialyzers. The best implementation was obtained from collodion with respect to fabrication, its dialysis effects, safe sterilization and because it can be obtained in any geometric shape” [2].

Unfortunately, Haas’s pioneering efforts in human dialysis were never acknowledged by the medical community. It was another doctor, Willem Kolff of Kampen, the Netherlands, who made the next big step in dialysis by constructing the dialysis machine, together with Henrik Berk.

The blood purification and dialysis procedure on animals was developed at John Hopkins University, Baltimore, by John Jacob Abel, Leonard Rowntree, and Benjamin Turner. In 1913 the researchers published an article describing “vividiffusion”—the removal of chemical substances from the blood stream of animals in the dialysis process [5]. In their paper from 1914 [6], the authors described a method when the blood taken outside a patient’s body was purified and transmitted back to its natural vascular circulation system in a continuous flow [3] through a path isolated from the air [2]:

“...Principle of the method is in connecting an artery of the animal by a cannula to an apparatus made from celloidin…in the form of tubes, immersed in a saline solution or serum and providing for the return of the blood to the animal’s body by another cannula attached to a vein… The blood leaving the artery flows through a perfectly sealed system and returns to the body within a minute or two without having been exposed to contact with the air or any chance of microbial infection, while the diffusible substances which it contains can pass out, more or less rapidly through the walls of the tubes. Coagulation of the blood is prevented by injection of “hirudin”…” [6].

Abel and colleagues named the device for dialysis “the artificial kidney’’ [2,3,6].

Willem Kolff, a physician, used a rotating drum kidney constructed by Henrik Berk, an engineer, for treatment of a patient in 1945 [7]. The treatment took as long as one week and the result was successful: the patient (a 67-year-old woman with acute kidney failure diagnosis) lived for over 6 years after the procedure (and died after an unrelated illness). This successful attempt confirmed the effectiveness of therapeutic method based on the use of “artificial kidney” [8] and hemodialysis principle suggested by Haas and Abel’s group. At that time, cellophane was used as a new filtering material in the dialysis tubes filled with blood [5].

Another breakthrough in hemodialysis was achieved by Swedish physiologist and inventor Nils Alwall of Lund University [9]. Nils Alwall’s artificial kidney was a superior device when compared to the one designed by Kolff since it not only allowed to purify the blood, but also to remove excess water after the dialysis procedure (ultrafiltration). The basic principle of ultrafiltration is to squeeze plasma water through filter membranes under pressure, after uremic toxins are removed during dialysis. The first patient (a 47-year-old man) was treated at the Lund hospital in September 1946. The 11-meter-long tubes that were used as filters for dialysis were made of cellophane–the same material used in the food industry at that time, e.g., for wrapping hotdogs.

Several technical problems appeared. For example, for the patients with chronical kidney disease it was difficult to use cannules made of glass. The problem was solved in 1960, when Belding Scribner together with surgeon Wayne Quinton of the U.S. created the arteriovenous shunt with two small Teflon outlets leading to the artery and the vein [1].

Another problem related to the first dialysis machine was the enormous space that the device was occupying. This has been improved by Frederik Kiil, a Norwegian physicist, who in the 1960s managed to create a cellophane dialysis filtering system which was much more compact due to the fact that only a small amount of blood taken from the body was used for circulation in the dialysis machine [1]. Cellophane membranes, most common in the 1970s, represented a convenient and relatively inexpensive solution for dialysis as the filters could be reused in the process.

However, cellophane membranes were mechanically unstable and leaked during the continuous dialysis process [3]. Instead, in late 1960s, cuprophane and cellulosic-based membranes were proposed to be used as blood purification filters [3]. In the 1970s, the importance of biological tolerance of blood-contacting material was brought into focus in clinical tests of extracorporal devices [3]. Among other physical properties, the hydrophobic-hydrophilic interactions of blood components with the filter membrane play a key role. Since the original cuprophane membranes were found to be non-biocompatible, further research in this direction continued [10,11,12].

Cellulosic membranes are hydrophilic polymers. In water, due to the polar nature of cellulose molecules, a hydrogel layer is formed on the membrane surface [3]. Accordingly, it was found that the hydroxyl groups on the membrane surface could be partially involved in the complement activation [3]. To minimize the activation of the complement cascades and to reduce leukopenia, the second generation of cellulosic-derived membranes was subsequently introduced for clinical use in the dialysis therapy. These improved materials were derived from cellulose substitutes such as cellulose acetate, cellulose diacetate and triacetate, as well as hemophane [3]. Later efforts were directed towards the surface modification of membranes with the aim of improving their biocompatibility, with the complement system activation becoming the “golden standard” of the biocompatibility check [3]. Simultaneously, in the 1970s, the first synthetic membranes for hemodialysis using polyacrylonitrile (PAN)-based polymer (originally hydrophobic) were produced by Rhône Poulenc in France, after the experimental observation of enhanced permeability of membranes to higher molecular weight compounds [13,14].

Currently, both artificial polymer and cellulosic modified membranes are used in dialysis therapy. This paper more closely examines the modeling of water transport across polymeric dialysis membranes in Section 5 and Section 6.

Quantification of dialysis is the result of nearly seventy years of joined efforts of scientists working in practical medicine, chemistry of polymers, physics and engineering, mathematics. A review on the quantification of dialysis from a historical perspective can be found in [5].

### 2.2. Peritoneal Dialysis

Peritoneal dialysis is an alternative blood purification method applied in cases of severe chronic kidney disease (CKD), with peritoneum of the patient’s abdomen used as the dialysis filter. When compared to hemodialysis, peritoneal dialysis is a less costly alternative. The first successful peritoneal dialysis was performed on animals by a German researcher, Georg Ganter in 1923. After this remarkable achievement, significant contributions to the method’s development were made in the U.S., first by a Wisconsin trio of Wear, Sisk and Trinkle, who in 1936 suggested a system for continual peritoneal dialysis, and then by Tenckhoff, who developed a special bacteriologically safe abdominal catheter [15].

CKD therapy encompasses a significant amount of patients, with approximately 1.6 million patients in the world currently being treated with hemodialysis and approximately 200,000 patients treated with peritoneal dialysis. In Sweden (according to the 2009 statistics), 2760 patients were treated with hemodialysis, while 839 with peritoneal dialysis [9].

## 3. Transport of Water across Natural Membranes

### 3.1. Fluid Pathways in the Organism

Transport of water across natural biological membranes or filters is attributed to different mechanisms: either via special water channels called aquaporins or related to the direct transportation of water through the pores in the membrane wall.

Kidneys are the natural filters in the organism. There, in the first stage of the urine formation in the process of blood ultrafiltration, the crucial role is played by glomerulus. The glomerular capillary walls form the filtration barrier for the blood plasma sieving proteins and other macromolecular components. Damage of the glomerular filtration barrier (GFB) may cause chronical kidney disease (CKD) or lead to the kidney failure. Impaired GFB function must be substituted with renal replacement therapy (RRT) or dialysis. Understanding of the mechanisms of filtration involved in GFB sieving may essentially help glomerular repair therapies and facilitate dialysis improvement.

Peritoneum forms a natural biological membrane where microvessels are distributed in the peritoneal tissues. In RRT, these peritoneal microvessels serve as dialyzing capillaries. In the peritoneal dialysis (PD), the capillaries permit slow transport of fluid and solutes. Continuous diffusive removal of small solutes and convectional removal of large solutes from the organism across peritoneal membrane pertain dialysis process.

The most important features of glomerular and peritoneal systems are summarized in the following two sections.

### 3.2. The Glomerular Filtration Barrier (GFB) in Kidneys

#### 3.2.1. GFB Structure in a Nutshell

**Figure 1:**
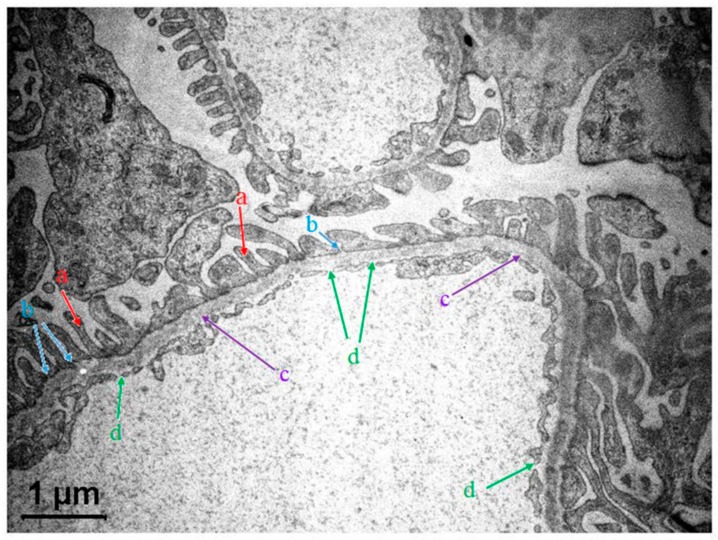
An electron microscopy of the glomerular filtration barrier (GFB) layer structure with podocytes (a, shown with red arrows), slit diaphragms (b, blue arrows), glomerular basement membrane (c, violet arrows) and endothelial fenestrae (d, green arrows).

Traditionally, the glomerular filtration barrier (GFB) was considered as a three-layer structure, composed of vascular endothelial cells, glomerular basement membrane and outer epithelial cells (Figure 1). New experimental data revealed the existence of two more layers [16]—the endothelial glycocalix and the sub-podocyte space [16,17]. The role of podocytes in the GFB maintenance and the importance of glycocalix is considered in detail in [18]. The highly debated statement on the porous structure of the glomerular endothelium can be formulated as following: the glomerulus is not just a “leaky barrier” [18].

In its turn, the glomerular basement membrane (GBM) comprises three fibrous layers: the inner layer, central lamina densa and outer lamina externa. The three-layer GBM provides mechanical support to endothelial cells and serves as a molecular sieve (the latter statement has been questionable in the GFB research [19]). The barrier function of GF has been associated with slit diaphragms of podocytes. There is experimental evidence that the special structural features and composition of GBM also contribute to the restriction of proteins’ (albumin) passage [20].

The podocytes are considered as an additional filtration system. The charge selectivity properties of podocytes filter, attributed to the extracellular glycocalix holding negatively charged sialic acids, have been discussed in a number of recent publications [21,22]. It was found [21] that the area between foot processes of the epithelial podocytes is sealed by filters or slit diaphragms. Electron tomography data displays a molecular architecture of slit diaphragms as a network of protein strands containing nephrin [23].

In contrast, the separating space between endothelial cells was shown to contain no diaphragm. Glomerular endothelium has a fenestrated structure, which also contributes to the transport of water and solutes. Simultaneously, glomerular endothelium fenestrae are considered to be diaphragm-less. These pores seem do not efficiently restrict the leakage of plasma proteins [21]. At this point, one should bring attention to the following opinion discussed in [24]. The authors [24] note that the glomerular endothelial cell fenestria are similar to the filtration slits of podocytes but not studied in detail. However, this may be important since the glomerular filtration rate is dependent on the surface area covered with these structures. An important topic debated in the paper [24] concerns the “glycocalix-in-the-fenestra” physiological role.

The GFB is a highly dynamic structure [25,26]. Despite the large number of biological data on GFB, the filter dynamics as well as involvement of its components and their correlated movement remain to be uncovered. The motility of the podocytes in the Bowman’s capsule has been recently tracked in vivo [26,27] in mouse models by multiphoton microscopy. This new imaging technique revealed simultaneous migration of fluorescently labeled GFB cells and spontaneous formation of cell clusters during the imaging of a normally functioning kidney [26,27]. These advanced research facilities may provide important answers to the questions regarding what kind of mechanical motions are involved in the filtration function.

Among the most successful methods for GFB structural studies one should also mention stereology and image analysis applied for the quantification of MRI results of the kidney [28,29], including the glomerular number and size distribution [29].

#### 3.2.2. Modeling Glomerular Sieving

The great efforts were directed to clarify the sieving mechanism in glomerular filtration [30,31,32,33,34,35,36,37,38,39,40,41,42,43,44,45,46,47,48,49,50,51,52,53,54,55,56]. Proteins and polysaccharides have been traditionally used as molecular probes for GFB selectivity studies [47]. It is well-established that the glomerular barrier freely filters small solutes however retaining large and negatively charged plasma proteins [48]. The diffusion and convection of polysaccharides, such as Ficoll and dextran, across the GFB is a traditional method for testing glomerular permselectivity [53]. It was shown that the effects of molecular size, shape, charge and deformability can also be studied with the help of these molecular probes [53]. Glomerular sieving of a number of neutral polysaccharides has been investigated in [47]. In comparison with proteins, polysaccharides demonstrate beneficial characteristics, for example, wider size spectrum of probes that can be used in a single experiment [47]. Then, the glomerular sieving coefficients obtained for these polymers can be compared with the characteristics of proteins, to resolve the effects of the geometrical size on glomerular barrier permeability (permselectivity). Several findings have attracted experimental and theoretical attention, in particular, how to include the significant conformational flexibility of the molecular probes.

A distributed two-pore model suggested in [55] has been successfully applied to the description of water and solute transport in biological membranes of different organs and recently, to artificial dialyzer membranes [50,51]. In particular, the model has been applied to the analysis of the experimental data on glomerular sieving of Ficoll. The authors noted that, typically, in the models applied to the filtration of solutes over a porous barrier, it is assumed that the molecules behave as rigid spheres. However, the experiments show that flexible polysaccharide molecules such as dextran or Ficoll, used as sieving probes, are hyper-permeable across the GBF. The calculations of the model provides the theoretical support for the idea that the flexible macromolecules’ sieving mechanism is different in comparison with the rigid spheres approach. In the model, the glomerular capillary wall is represented as a barrier with two populations of small–and large-pore populations [55]. The calculations of the model provide the theoretical support to the idea that sieving the mechanism of flexible macromolecules is different in comparison with the rigid spheres approach. The variance in distribution of pore sizes has been attributed to the molecular “flexibility” of Ficoll, assuming that the true variance of the pore system is lower than the one obtained using flexible probes.

Molecular probes studies also allow researchers a detailed quantitative analysis of the electrostatic effects [54]. In this approach, the glomerular filtration barrier is modeled as a charged fiber matrix that separates charged and neutral Ficoll molecules [54]. To explain the measured difference in the glomerular transport between the neutral and charged (anionic) form of Ficoll, calculations of surface charge density and simulations performed for the solutes with charge density similar to that of albumin (−0.022 C/m^2^ (albumin), −0.035 C/m^2^ (Ficoll approximation)) were carried out in [54]. Correlation of the theory with data analysis demonstrates that the electrical charge makes a moderate contribution compared to the size and conformation which seems to be more important for the filtration of macromolecules.

Experimental studies of size-selectivity (sieving) of natural glomerular filters [52,53,55] and synthetic dialyzing membranes [51] by using molecular probes (Ficoll, dextran and proteins such as albumin or myoglobin) revealed the important effects of molecular size, shape, charge and deformability of the probes. It was shown that, in comparison with the globular proteins, dextran (polysaccharide) and Ficoll (a highly cross-linked copolymer of sucrose and epichlorohydrine) are more “permeable” [51]. This experimental finding has been attributed to the flexibility and “molecular extension” of polysaccharides, which exhibit an open, deformable structure [51]. The penetration of these molecular probes through the pore thus, should be considered as a transport of flexible polymer molecules rather than rigid spheres. In studies of charge selectivity in the glomerular filtration, the electrostatic effects (for the charged probes) should be compared with the flexibility factors.

#### 3.2.3. Debates on Albumin Sieving

Because of the complexity of GFB structure and possible controversial interpretation of the experimental data, there is still no unified view or last judgment on how the biological components of the kidney filter contribute to the protein sieving [20,40].

The sieving of albumin is one of the most important parameters for proteinuria characterization [40]. The measurable parameter is the glomerular sieving coefficient (GSC) defined as the filtrate-to-plasma concentration of the protein [20,40]. Discussion around albumin filtration–the glomerular vs. tubular origin, has been reviewed in a number of publications [20,40,41,42,56]. In particular, in [40], Haraldsson and Deen (con) strongly criticized the assumption of tubular reabsorption of proteins and mentioned that the massive experimental data provide the evidence that glomerular barrier normally is both size–and charge–selective. However, the defects in a damaged GBF may cause the albumin leakage leading to increased concentration of the protein. In the same paper [40], another participant in debates, Comper (pro), provides a support to the alternative view. Haraldsson and Deen [40] also mentioned that the experiments with the radiolabeled albumin studied in [46], as a proof of albumin retrieval, have been misinterpreted. Recent theoretical analysis in [56] reveals that the electrophoresis does not significantly contribute to albumin filtration across GFB.

### 3.3. Comparing Glomerular and Peritoneal Transport

By comparing transport across glomerular and peritoneal barriers, the relevant question arises what is the difference between these two systems?

**Figure 2:**
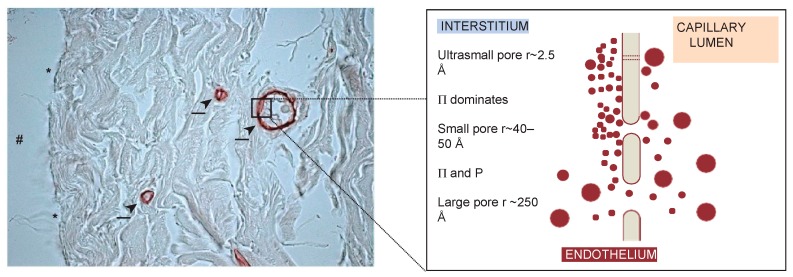
Microscopic structure of the peritoneal membrane and the three-pore model (TPM). (Figure 2 is reproduced from [59] with permission). **To the left**: Micrograph of the cross-section of the human parietal peritoneum stained for the water channel aquaporin-1 (AQP1). The peritoneal membrane contains three components: a layer of ciliated mesothelial cells (*), with microvilli and apical protrusions into the peritoneal cavity (#); the interstitial tissue consisting of bundles of collagen and mucopolysaccharides; and a dense network of capillaries, blood vessels, and lymphatics. During peritoneal dialysis (PD), the microvascular endothelium (arrows, stained in red) represents a functional barrier for the transport of solutes and water from the blood of the patient to the dialysate that has been instilled in the peritoneal cavity. **To the right: (inset)** The endothelium lining the peritoneal capillaries can be functionally described within the three-pore model (TPM). The small pores (radius ~ 40–50 Å), located between the endothelial cells, account for ~90% of the peritoneal ultrafiltration coefficient and 99.5% of the total pore area available for solute transport. The large pores (radius ~ 250 Å), thought to correspond to interendothelial gaps occupying <0.5% of the total pore area. The ultrasmall pores (radius ~ 2.5 Å) are the only ones to be located in the endothelial cells. (Caption to the Figure 2 is adapted from [59] with permission).

In general, the glomerular filtration barrier displays more complex morphology than the peritoneal membrane [57] (however, the morphology of peritoneum shown in Figure 2 reveals the tortuosity of inter-endothelial clefts and network of capillaries embedded into the interstitium [58,59]). Also, the highly dynamic GFB structure has been recently confirmed in [25,26,27] studies. The uniformity of GFB is another remarkable structural feature. In this aspect, glomerulus is more similar to the artificial membranes than peritoneum [57]. Rippe and Davies noticed that, due to GFB uniformity, filtration across the glomerular capillary wall (GCW) better conforms to the pore theoretical analysis [57]. GFB is far less leaky than peritoneum barrier. With respect to the protein sieving, GFB is more size–selective and discriminates polymers according to their shape and flexibility [57]. The GFB also demonstrates charge selectivity. However, this is considered now as a much less influential factor.

For quantitative description of the transport across the peritoneal capillary wall, the so-called three pore model (TPM) has been developed [53,58,59], which plays an important role for the understanding of PD mechanism. Section 3.4.2 provides a brief account for the transport of water across the capillary endothelium of the peritoneum described within the TPM approach. The ultrasmall pores involved in the transport of water in peritoneal membranes are associated now with aquaporins as the “third pores” [59,60,61]. The following section introduces the readers to these crucial-for-life proteins.

### 3.4. Aquaporins

#### 3.4.1. “Water-Only” Nanopores

Before the discovery of aquaporins it has long been assumed that water can freely penetrate through biological membranes. Then, the existence of family of water channels’ has been proved: it was shown that some membranes of cells are more after permeable than other types of cells as well as pronounced osmotic reaction in comparison with diffusional water permeability [62,63,64,65,66,67,68,69,70,71,72,73,74,75,76,77,78,79,80,81,82,83,84,85,86,87]. This enhanced osmosis water transportation across membranes suggested the existence of special pathways involved in water transfer. Structure of aquaporins and molecular dynamics studies revealed that water penetrates in a single-file transport manner through a nanopore in protein monomer [66,67]. The selectivity of water molecules penetration is controlled by steric and electrostatic forces [71].

Aquaporins belong to the family of integral membrane proteins forming permeable for water pores in cell membranes. Aquaporin 1 (AQP1), the first found aquaporin protein, plays an important functional role in the kidney. Originally, AQP1 water channel was discovered in red blood cell membranes [71,72,73,74,75,76]. AQP1 is expressed in proximal tubules, thin descending limbs of the loop of Henle which are involved in reabsorbing of the most of filtered water (90%) in the kidney [77,79] (not regulated by vasopressin). AQP1 was also found in red blood cell membranes, in lungs, vascular endothelial cells and sweat glands among others. Aquaporin 2 (AQP2, water channel regulated by vasopressin) was found in collecting duct cells of the kidney. The AQP2 channels regulate the reabsorption of the remaining fluid [77]. Other important aquaporins are AQP4 (expressed in brain), AQP5 (found in secretory glands and lungs), AQP7 (the aquaglyceroporin responsible for glycerol release during starvation) and AQP9 (found in liver tissue) [71].

Many biological functions of cells are regulated by the aquaporins (AQPs) [60,61,62,63,64,66,67,68,69,70,71,77,78,79,82]. The involvement of AQPs in fluid transport in cells facilitates passive water transport when large osmotic gradients are applied. It was shown that AQPs also play a key role in active fluid absorption and secretion near isosmolar processes [67]. In the collecting duct epithelial cells, water transport is related to vasopresin regulation [68,69,77]. In the kidneys, collecting duct aquaporins are involved in the urine concentration mechanism, facilitating osmotic water transport. In various cells, AQPs facilitated water transfer across membranes plays a key role in a range of physiological functions of the cells [70,71,72,73,74,75,76,77,78,79]. The pathology in AQPs functioning may lead to the so-called human aquaporin diseases [67,77]. Studies of AQP1s demonstrated that these water channels are involved in regulation of water and ions in blood vessels of different organs [75,76,77]. Early investigations of swelling processes on isolated cells [78] and on reconstituted liposomes [79] confirmed that AQP1 proteins facilitate osmotically driven water flows. Also, the AQP1 knockout mice initial studies [80] show that the osmotically caused water transport in peritoneum of AQP1¯¹¯ animals was essentially diminished in comparison with wild ones [73].

The regulation of water flow in and out of cells can be influenced by osmotic gradients across the membrane. In Section 3.4.2, the osmotic water transport across the peritoneal membrane and the involvement of AQP1s are briefly discussed.

#### 3.4.2. Peritoneal Dialysis: Ultrafiltration, Osmotic Water Transport and AQP1s Involvement 

During the PD session, the osmosis-induced pressure across the peritoneal membrane is used to take away excess water and solutes [78,79]. The efficiency of osmotic water transport and the quantification of free water are the most important processes in PD.

The quantification of free water in peritoneal dialysis during the peritoneal equilibration test (PET) is a generally accepted method for studying solute transport and ultrafiltration in PD [88,89,90,91,92,93,94,95,96,97]. In the peritoneal equilibration test, the sieving of sodium (which is considered associated with a hypertonic glucose solution) allows researchers to evaluate aquaporin-related transport of water [96]. In the standardized PET [88,89,90,91,92,93], free water transport (FWT) and small solute transport across the peritoneal barrier can be calculated after a 60-min dwell with 3.86% glucose by analyzing the sodium transport kinetics. (For the detailed description of the standard PET procedure see, for example, in the paper [89]). For FWT calculations, the intraperitoneal volume should be measured [88,89,95,96]. The UF is defined as a ratio of the transported sodium amount to the plasma sodium concentration [89]. The FWT is calculated as a difference between the total UF value after one hour and the small-pore transport value [89]:

(1) $$FWT=UF_{Total\;after\;1\;hour}-UF_{small-pore\;transport}$$

During PD, crystalloid osmotic pressure gradients induce transport of fluid which incorporates transport across small pores and free water transport [92,94]. The FTW is considered as related to AQP1 pathways [74]. The input of both pathways can be quantitatively determined by comparing the kinetics of water and sodium transport [88,89,93,95].

The existence of the ultrasmall pores associated with AQP1s in the peritoneal membrane has been suggested for the explanation of the osmotic effectiveness of glucose and the sodium sieving (which is the phenomenon of rapid fall in dialysate sodium concentration during a dwell with a hypertonic glucose solution) [78]. To study the role of osmotic gradients, a different concentration of glucose solution could be used [93,96]. By using radio-labeled albumin and low concentration (1.36%) glucose solution, Asghar and Davies determined changes in the intraperitoneal sodium concentration and, then, evaluated the transperitoneal clearance of sodium [96]. Fluid pathways and reabsorption in the peritoneum within the TPM concept have been experimentally studied in [96] at lower concentration of glucose than suggested by LaMilia and co-authors in [93]. This work deserved a comment by Rippe and Venturoli [97] on back-filtration of fluid through the small pores in view of the three pore model. Recently, the ultrafiltration and osmosis have been studied in mice by using a fluorescent probe (Alexa Fluor-555 albumin) as a tracer [79]. A comparison of the results with radio-labeled albumin measurements revealed the reliability of the fluorescent technique to quantify the osmotic water transport in vivo [79].

The identification of AQP1 as a most relevant to UF component, brought a new understanding of water transport processes across peritoneal membrane [73,74]. Ni and co-authors [74] in mice lacking AQP1 research reported on 50% UF decrease which was accompanied by the restriction in sodium sieving in peritoneal capillary endothelium. Conversely, it was shown that the induction of AQP1s in the peritoneal capillaries (by corticosteroids) has stimulated water transport and UF without changes in the osmotic gradients and transport of small solutes [74]. These results suggest that the AQP1s are responsible for the sodium sieving and mediate half of UF during the hypertonic dwell. The regulation of AQP1 expression with the aim to increase the ultrafiltration has been a topic of a large number of experimental efforts (see, for the review, [59,73,81,82,83]).

#### 3.4.3. Debates

The osmoregulatory mechanisms in cells are complex and the correlations between many factors involved in these processes are not well studied. An example is recently discovered coupling between the transfer of water across aquaporins and sodium transportation by molecular machines (a family of special proteins, secondary transporters) across the cell membrane [84]. At the moment, there are several opinions on the AQP1 role and its involvement in the UF regulation. The alternative mechanism compared to the above mentioned has been reported in a number of current publications (see, for example, [85,86,87]).

The mechanism of transperitoneal exchange received close attention and numerous discussions around osmotic water transport and the role of aquaporins [73,74,78,79,81,98,99,100,101,102,103,104,105,106]. In particular, Flessner in the comment [81] to the paper by Ni and co-authors [74] discussed the potential influence of the glycocalix on peritoneal transport. The author also considered a role of endothelial glycocalix in abnormal cells and argued that the defects in damaged glycocalix could form pathways for the fluid and solutes transport. The author recollected the so-called “pore-matrix theory” [107] and referred to the experiments [108] where it was shown that inflammation can lead to the glycocalix alteration. The latter, according to the author’s opinion, may increase the permeability of the endothelium to solutes such as glucose and sodium that, in its turn, influences the UF.

## 4. Fundamental Thermodynamic Relations for the Transport of Fluid and Solutes in Dialysis

Theoretical and experimental studies of transport across membranes constitute a broad area of research, encompassing issues ranging from thermodynamics of water and solute flows to the use of modern computational tools exploring water for micro– and nano-scale modeling of water and ion transportation.

Traditional models for water transport account for the osmotic effects formalism, developed by Kedem and Katchalsky in their early works [109,110,111]. Their theory is based on non-equilibrium thermodynamics of irreversible processes. Another fundamental concept used for the description of transport phenomena in various media is Onsager’s reciprocal relations. Due to the importance of these formulations we shall present their overview in a separate theoretical physics subsection below.

### 4.1. Nonequilibrium Thermodynamics of Irreversible Processes

The basics of nonequilibrium thermodynamics has been formulated by Lars Onsager, including reciprocal relations for the kinetic coefficients in transport phenomena described within the generalized forces–fluxes linear equations [112]. In the thermodynamics of irreversible processes, for n fluxes and all driving forces, respectively, a system of equations can be written as follows [113]:

(2) $$J_{i}=L_{ij}X_{j},\;j=1,\ldots n$$

Here $J_{i}$ is a flux, $X_{j}$ is a conjugated force, and $L_{ij}$ is the appropriate kinetic coefficient. The principle of symmetry of kinetic coefficients, or Onsager’s principle [112] states that

(3) $$L_{ij}=L_{ji}$$

In general, the symmetry of the kinetic coefficients or Onsager’s principle reflects the deep-lying internal symmetry in the relaxation of the system not far from the thermodynamic equilibrium [114]. In terms of thermodynamically conjugate variables one can write:

(4) $$Y_{i}=-\frac{\partial S}{\partial y_{i}}=\beta_{ik}y_{k}$$

(5) $$y_{i}=-\frac{\partial S}{\partial Y_{i}}$$

where the defined quantities correspond to the entropy of the system, $S\left(y_{1},\ldots .,y_{n}\right)$, as a function of n physical variables $y_{1,}y_{2},\;\ldots .,y_{n}$ describing the system [114]. With respect to the time reversal transformation, the relations in Equation (3) are valid only if physical quantities in Equations (4) and (5) both change sign (for example, when these variable are proportional to the velocities in the system). In the case when only one of the quantities $y_{i}$ change sign under time reversal while the other $y_{k}$ remains unchanged, the Onsager’s principle of the symmetry of kinetic coefficients gives [114]:

(6) $$\beta_{ik}=-\beta_{ki}$$

In particular applications, the linear relations in Equation (2) together with Equation (3) form a basis for the fluxes-forces analysis in transport processes. Transport of water and solutes across filter membranes and the thermodynamic approach known as Kedem–Katchalsky theory [109,110,111] is a special case of general relations (2) and (3) where the kinetic coefficients obey the Onsager’s principle:

(7) $$J_{1}=L_{11}X_{1}+L_{12}X_{2}$$

(8) $$J_{2}=L_{21}X_{1}+L_{22}X_{2}$$

Within the phenomenological theory, the relations between the total volume flow of transported fluid and the exchange flow are [113]:

(9) $$j_{V}=L_{p}∆P+L_{pD}∆\Pi$$

(10) $$j_{D}=L_{D}∆\Pi +L_{pD}∆P$$

with the Onsager’s reciprocal relation for the coefficients:

(11) $$L_{pD}=L_{Dp}$$

The physical meaning of the kinetic coefficients is introduced in the Kedem–Katchalsky theory of transport of water and solutes across filtering membranes (see Section 4.2).

For a coarse nonselective membrane, in the absence of the hydrostatic pressure difference, ΔP=0, the volume flow vanishes:

(12) $$j_{V}=L_{pD}∆\Pi =0$$

When the osmotic pressure is absent, ΔΠ=0 the exchange flux $J_{D}=0$ and the ultrafiltration of nonselective membrane caused by the hydrostatic pressure only is absent:

(13) $$j_{D}=L_{pD}∆P=0$$

The selectivity of the membrane is introduced via assumption of the semipermeable membrane. For the latter, the osmotic flow and ultrafiltration play role which expressed in the non-zero cross coefficient, $L_{pD}$. In ideal semipermeable membranes,

(14) $$j_{D}=-j_{V}$$

and both volume and exchange fluxes are due to the water flow only [113] characterized by a single kinetic coefficient, the hydraulic conductivity $L_{p}$.

For the description of transport of dissolved substances, it is convenient to introduce a flow of solvent rather than the exchange flow. For dilute solutions, Kedem–Katchalsky theory suggested for the flow of solvent $j_{s}$

(15) $$j_{s}=\left(j_{V}+j_{D}\right)C_{s}$$

where $C_{s}$ is the (small) volume concentration of the dissolved compound.

The reflection coefficient σ introduced in the theory, (also called the Staverman reflection coefficient), is defined as following:

(16) $$\sigma =-\frac{L_{pD}}{L_{p}}$$

A system of two phenomenological equations incorporating both kinetic coefficients [113]:

(17) $$j_{V}=L_{p}\left(\Delta P-\sigma RT\Delta C_{s}\right)$$

(18) $$j_{s}=\chi RT\Delta C_{s}+C_{s}\left(1-\sigma \right)j_{V}$$

where

(19) $$\chi =\left(L_{D}-L_{p}\sigma^{2}\right)C_{s}$$

Equations (17)–(19) provide a complete thermodynamical description of transport of water and a single uncharged solute across semipermeable membrane.

#### Starling Equation

One can use the approach of non-equilibrium thermodynamics to describe a process of fluid filtration across the capillary wall (considered as a semi-permeable membrane) assuming that there is a difference in protein concentration between the plasma and tissue (the so-called, oncotic pressure difference). In the system of kinetic Equations (17) and (18), Equation (17) can be presented in the following form:

(20) $$J_{V}=L_{p}S\left(\Delta P-\sigma \Delta \pi \right)$$

where $L_{p}$ is the hydraulic permeability of the capillary wall, S is the surface area, $∆P=P_{c}-P_{i}$ is the difference between the capillary (index “c”) hydrostatic pressure and interstitial (index “i”) pressure. The osmotic (oncotic) pressure $\Delta \pi =\pi_{p}-\pi_{i}$ is due to the difference between plasma protein oncotic pressure and the interstitial oncotic pressure, σ is the Staverman (reflection) coefficient. The ratio $j_{V}=J_{V}/S$ describes the fluid filtration flux across the capillary wall per unit area.

Equation (20) is known as a Starling equation, which a classical kinetic equation of capillary filtration.

### 4.2. Thermodynamics of Membrane Transport

Kedem-Katchalsky classical model based on the irreversible thermodynamics approach (and Onsager’s relations) describes the flow of solvent, $J_{V}$ and solute $J_{S}$.

For the semipermeable membrane, the solvent flow is given by the Equation (17). This equation may be generalized for the case when a nonpermeant solute is added to the solution [113]:

(21) $$J_{V}=L_{p}S\left(\Delta P-RT∆C_{n}-\sigma RT\Delta C_{S}\right)$$

Here $C_{n}$ is the concentration of a nonpermeant solute (i.e., its reflection coefficient σ is 1). For permeating solutes, the reflection coefficient 0<σ<1 [113].

For the flow of solute, $J_{S}$ across semipermeable membrane, the Kedem-Katchalsky model states [109,110,111]:

(22) $$J_{S}=J_{V}\left(1-\sigma \right)\bar{C}+PS\Delta C$$

In this equation $\bar{C}$ is the mean intramembrane concentration of solute, ∆C is the gradient of solute concentration and P is the solute permeability coefficient $P=\frac{D_{S}}{∆x}$, which is a ratio of the diffusion coefficient $D_{S}$ to the diffusion distance ∆x. The solute diffusion coefficient $D_{s}$ is given by the relation:

(23) $$D_{s}=\frac{RT}{6\pi N_{A}\eta a_{s}}$$

Here, R is the gas constant, T is the temperature (in Kelvin degrees), $N_{A}$ is the Avogadro’s number and $a_{s}$ is the Stokes-Einstein radius of solute.

In the early publications [115,116,117], Equation (22) has been employed for evaluation of the capillary permeability to macromolecules (but using the simplifying assumption σ=1).

General Equations (21) and (22) have been applied to the analysis of membrane transport across various biological filters, from model semipermeable membranes to the complex filtration systems such as kidney filtration barrier or transvascular walls [33,35,55,118]. In the latter system, the solute gradient $∆C\left(=C_{p}-C_{i}\right)$ value is the difference between solute concentration in plasma, $C_{p}$, and in interstitium, $C_{i}$, respectively [33,55]. The mean solute concentration is, in general, a function of the fluid flow $J_{V}$ and of the product of the membrane solute permeability, P, and the membrane surface area S.

The integration of Equation (22) over the membrane barrier [33,55] gives the following expression for the solute clearance, K, which is the solute flux, $J_{S}$ divided by $C_{p}$ value:

(24) $$K\equiv J_{S}/C_{p}=J_{V}\left(1-\sigma \right)\frac{1-\left(\frac{C_{i}}{C_{p}}\right)e^{-Pe}}{1-e^{-Pe}}$$

The Péclet number ($P_{e}$) is defined as a ratio of the solvent flow and to the capillary diffusion capacity (the “convection –to–diffusion ratio”) [33,55]:

(25) $$P_{e}=J_{V}\left(1-\sigma \right)/PS$$

From Equation (24), the capillary diffusion capacity, can be calculated for any given concentration ratio $C_{i}/C_{p}$ and for a given type of solvent flow (for example, a lymph flow), $J_{V}$ and the reflection coefficient of the membrane, σ [33]:

(26) $$PS=J_{V}\left(1-\sigma \right)/ln\left[\frac{\frac{C_{i}}{C_{p}}\sigma }{\frac{C_{i}}{C_{p}}-\left(1-\sigma \right)}\right]$$

When the interstitial space is large and the solute concentration in there is low, respectively, one can get a simplified expression for the solute clearance from Equation (24):

(27) $$K=J_{V}\frac{1-\sigma }{1-e^{-P_{e}}}$$

For the steady state, the general equation for the clearance in Equation (24) may be rewritten as a sum of two terms [33]:

(28) $$K=PS\frac{P_{e}}{e^{Pe}-1}\frac{C_{p}-C_{i}}{C_{p}}+J_{V}\left(1-\sigma \right)$$

In Equation (28), in case of a large Péclet number, the first (“diffusive”) term vanishes so the second (“convective”) term dominates in the process. This means that the ‘diffusive’’ transport is negligible at high volume flow values so the solute clearance is described by the simple formula:

(29) $$K\approx J_{V}\left(1-\sigma \right)$$

For the cylindrical pores, by applying Poiseuille’s law, one can calculate the tissue capillary hydraulic conductance:

(30) $$L_{p}S=\frac{A_{0}}{8\eta \Delta x}\left(\alpha \cdot r^{2}\right)$$

In Equation (30), $A_{0}$ is the total cross-sectional pore area, η is the viscosity of water and r is the radius of a cylindrical pore. In Equation (30), Δx denotes a unit diffusion path length (a thickness of the membrane wall for cylindrical pores).

Equations (21)–(30) represent main thermodynamic relations for the macroscopic description of the transport processes in membranes.

### 4.3. Two-Pore Membrane Model

One of the popular theories in dialysis is the so-called two-pore model [33], which combines the general non-equilibrium thermodynamics and kinetics of transmembrane transport in order to analyze the convection and diffusion processes. The two-pore model has been used to describe permeability and transport phenomena in blood capillaries and different organs [119,120,121,122], glomerular sieving [35,47,118] and, recently, the analysis of transport across synthetic dialyzing membranes [51]. The basic results obtained within the two-pore model are briefly summarized in the following two sections.

#### 4.3.1. Classical Two Pore–Model

Classical two-pore model considers transport of water and solutes across a (capillary) wall through two types of pathways, different in size, i.e., large and small pores of fixed diameter [33,96,123,124,125]. In this relation, the partitioning of fluid fluxes among transmembrane pathways may be considered within the homoporous models (for the studies of steady-state protein flux analysis, see [115,116,117,119,121,125,126,127] or heteroporous membrane models [95,125].

For the transport of fluid across the heteroporous membrane, one can write the generalized equation of the equation for m different-in-size pores [33]:

(31) $$J_{V}=\sum_{i=1}^{m}J_{v,i}=\sum_{i=1}^{m}\alpha_{i}L_{p}S(∆P-\sum_{j=1}^{n}\sigma_{i,j}∆\pi_{j})$$

Equation (31) describes the partitioning of fluid fluxes in porous membrane, where $\alpha_{i}$ and $\Delta \pi_{j}$ refer to the fractional hydraulic conductance related to a given pore type (index “i”) and the osmotic pressure difference due to solute “j”, and index “n” denotes the number of solutes. For two sets of pores, large and small in size, the reflection coefficient σ in (31) is a weighted sum of large (index “L”) and small (index “s”) pores [33]:

(32) $$\sigma =\varphi_{s}\sigma_{s}+\varphi_{L}\sigma_{L},\;\varphi_{s}+\varphi_{L}=1.$$

In Equation (32), $\varphi_{L}$ denote the fraction of fluid coming through the larger pores, $\varphi_{L}\equiv J_{V_{L}}/J_{V}$ (where $J_{V}$ is the glomerular filtration rate, or GFR, $J_{V_{L}}$ is the large-pore filtration), and $\varphi_{s}$ is the fraction of fluid crossing the filtration barrier through the smaller pores.

The steady-state transport of solute across the membrane is [33]:

(33) $$J_{solute}=J_{diffusion}+J_{convection}=-DS\frac{dc}{dx}+J_{V}\left(1-\sigma \right)c$$

The first term in Equation (33) describes the Fick’s diffusion while the second term is proportional to the volume flux $J_{V}$ (or the glomerular filtration rate, GFR) and concentration c(x), describes the convective transport.

It is convenient to introduce a model parameter, θ, the membrane sieving coefficient, which is a ratio θ = $C_{P}/C_{i}$. The solute clearance is the product of volume flux, $J_{V}$, and the sieving coefficient, θ. Using Equation (24), one can deduce for the sieving coefficient:

(34) $$\theta =\frac{1-\sigma }{1-\sigma e^{-Pe}}$$

In the two pore model, the sieving coefficient can be written as follows [55]:

(35) $$\theta =\varphi_{s}\frac{1-\sigma_{s}}{1-\sigma_{s}e^{-P_{e}{}_{s}}}+\varphi_{L}\frac{1-\sigma_{L}}{1-\sigma_{L}e^{-P_{e}{}_{L}}}$$

In Equation (35), $\sigma_{s,L}$ and $P_{e_{s,L}}$ correspond to the reflection coefficients and Péclet numbers for the small-size-pore and large-size pore population, respectively.

Sieving coefficient θ and Péclet number $P_{e}$ are important characteristics which can be deduced from the filtration experiments and clearance data [50,52,53,54,55,123,126,127,128,129,130,131,132]. Typically, θ coefficients and $P_{e}$ numbers are plotted against Stokes–Einstein radius (see, for example, FITC-Ficoll and FITC-dextran compared to proteins sieving studies reported in [50]).

To test the predictions of the two-pore model, several filter membranes with the different model parameters (small and large pore sizes) have been simulated in the computer studies [33]. In this study, the small pore radius was chosen in the interval 43 Å < $r_{s}<$ 55 Å while for the large pores $r_{L}=250$ Å [33]. The two-pore model has been applied in analysis of the experimental data on albumin and some neutral proteins and Ficoll [50,52,53] sieving for glomerular filtration barrier in rat kidneys. In [53], the two-pore model and log-normal distributed plus shunt analysis of sieving curves has been performed by using 48.9 Å < $r_{s}<$ 50.3 Å and 136 Å < $r_{L}<$ 140 Å values for the small and large pore radii, respectively.

#### 4.3.2. Distributed Two–Pore Model

The central assumption of the “distributed” two pore model is that there are two types of pores (with large and small radii, r) and that pore size is distributed around most probable radius value $r_{M}$, for each type of pores. By using the log-normal distribution function, f(r) with the deviation ln(Σ) (the geometric standard deviation) [55],

(36) $$f\left(r\right)=\frac{1}{r\ln\left(\Sigma \right)\sqrt{2\pi }}exp\left\{-\frac{1}{2}{\left[\frac{\ln\left(r\right)-\ln\left(r_{M}\right)}{\ln\left(\Sigma \right)}\right]}^{2}\right\}$$

one can calculate corresponding solute flux (mean) parameters. For example, the reflection coefficients for each type of pores are given by the expressions [55]:

(37) $$\sigma_{S}=\frac{\int_{0}^{\infty }r^{4}f\left(r\right)\sigma_{h,S}\left(r\right)dr}{\int_{0}^{\infty }r^{4}f\left(r\right)dr}$$

(38) $$\sigma_{L}=\frac{\int_{0}^{\infty }r^{4}f\left(r\right)\sigma_{h,L}\left(r\right)dr}{\int_{0}^{\infty }r^{4}f\left(r\right)dr}$$

In Equations (37) and (38), indices “h, S” and “*h*, *L*” refer to the homoporous small-pores and large-pores reflection coefficients. Then, for the corresponding Péclet numbers one can get:

(39) $$Pe_{s,L}=\frac{J_{V_{s,L}}\left(1-\sigma_{s,L}\right)}{PS_{s,L}}$$

and for the permeability–surface area product [55]:

(40) $$P\cdot S_{s,L}=\frac{D\frac{A_{0_{s,L}}}{H}\int_{0}^{\infty }\frac{A}{A_{0}}\;\left(r\right)r^{2}f\left(r\right)dr}{\int_{0}^{\infty }r^{2}f\left(r\right)dr}$$

where A is the apparent (effective) pore area, thus the ratio $\frac{A}{A_{0}}$ describes the degree of restricted diffusion (according to [48]), $A_{0_{s,L}}$ is the fractional cross-sectional pore area of the small- and large-pore systems, respectively.

Direct calculations within the distributed two-pore model provide the following expression for the total cross-sectional area of membrane barrier [55]:

(41) $$\frac{A_{0}}{H}=\frac{N}{H}\int_{0}^{\infty }\pi r^{2}f\left(r\right)dr=N\frac{\pi }{H}exp\left\{2\ln^{2}\left(\Sigma \right)\right\},$$

where N denotes the (total) number of pores per unit area of membrane, H is the membrane thickness.

For the hydraulic conductivity, $K_{f}$, one can find [55]:

(42) $$K_{f}=\frac{A_{0}}{H}\frac{r_{M}{}^{2}}{8\eta }exp\left\{6\ln^{2}\left(\Sigma \right)\right\}.$$

Other thermodynamic parameters, such as permittivity-surface area coefficients and volume fluxes across the filtration barrier have been calculated in a similar way (for the details of calculations, see in [55]).

##### On the Choice of Log-Normal Distribution Function

The log-normal distribution function has been often exploited in the analysis of pore size distribution in a wide range of natural biological and artificial filter membranes (see, for the review, in [133]). The probability density function (Equation (36)) used in [55] is a standard log-normal distribution that describes a filter barrier with pores of varying radii r with a most probable value $r_{M}$ of the pore radius, (which is the radius at the maximum in the distribution). A slightly different log-normal distribution function (compared to Equation (36)) was used in [133] for the calculating the total number of membrane pores. It was noticed that the proper choice of mathematical form for the distribution function strongly depends on specific problem, i.e., what kind of transport processes are described and what is the physical meaning of parameters calculated in the model with a given distribution function [133]. For example, different forms of log-normal probability density function have been used in the analysis of membrane ultrafiltration and fouling [133].

### 4.4. Three Pore Model

#### 4.4.1. Three Pore Model (TPM) of Peritoneal Membrane Transport

Transport across peritoneal membrane can be described within the approach called a three pore model suggested by Haraldsson [134], Rippe [58] and co-workers [59,135] in 1990s. The peritoneal membrane is a composite three–layer material. It is generally accepted [136] that endothelium of the capillaries plays the most important role in peritoneal dialysis (PD). During PD, this layer serves as a barrier for water and solutes being transferred from blood to dialysate into the peritoneal cavity. Qualitatively, transport across this porous barrier can be described in the following way.

The small pores (with radius r~40–50 Å) between the epithelial cells fulfill about 90% of the hydraulic conductance ($L_{P}S$) of the peritoneal membrane. The ultrasmall pores (with radius r ~ 2.5 Å) in the endothelial cells contribute only 2% to the hydraulic conductance of membrane [76]. The large pores (r ~ 250 Å) viewed as interendothelial spacings which occupy less than 0.5% of the total pore area and amount to 5–8% of the hydraulic conductance value [76]. Within the three pore model (TPM) approach, the partitioning of fluid flows has been attributed to the balance between Starling forces [76]. In small pores, the hydrostatic pressure gradients are approximately balance each other out. In large pores, the hydrostatic contribution dominates over colloid pressure since the oncotic gradients are almost negligible there. The three-pore model is illustrated in Figure 2 (see Section 3).

Various osmotic agents affect the fluid removal at PD in different ways. For example, small osmotic molecules such as glycerol ($r_{SE}\;\sim$ 3 Å), have insignificant impact on the transport across small pores, acting on water-only pores instead. In contrast, the addition of glucose induces water flows that are equally partitioned between small and ultrasmall pores [76]. During PD, the osmotic effects of glucose cause the dilution of dialysate that can further lead to the reduction in sodium concentration in there (the so-called “sodium sieving” phenomenon) [76]. The shift in the redistribution of Starling forces then lead to the fluid reabsorption through the small pores from the peritoneal cavity to plasma [106].

Recently [61,75,76], the AQP1 proteins were identified as ultrasmall pores which can be described within the TPM theory. Comparison of the theoretical predictions of the TPM [58,75,134,135,137,138] with the experimental data on fluid transport across the peritoneal membrane [58,96,97,98,137] shows that this model adequately describes the pathways of peritoneal fluid transport. Recent publication of Zhang and co-authors have confirmed the crucial role of endothelial AQP1s during peritoneal dialysis and provide a direct experimental evidence for the functional relevance of the TPM predictions [61].

Recent developments in PD practice [75,96,139] demand further research and modeling. Since peritoneal dialysis conditions are determined by a delicate interplay between diffusion, convection and osmosis water transport though the peritoneal barrier [75,76], the improved or automated PD implicates the need for more complex analysis. Recently, the extended theory based on three-pore model (TPM) has been elaborated in [140]. The model [140] has also been employed for the prediction of the ultrafiltration profiles in PD for various osmotic agents. In the next step, calculated ultrafiltration profiles [140] have been used to estimate the UF for a specific polymer (an osmotic agent) to improve fluid removal.

#### 4.4.2. Extended TPM and Applications to the Automated Peritoneal Dialysis

In a recent publication [141], extended thee pore model (TPM) was used to optimize patients’ treatment with automated peritoneal dialysis (APD).

APD is the process of peritoneal dialysis with the aid of a mechanical cycler with variable rates of dialysate flow. The most difficult task in APD modeling is time-dependence of dialysis parameters at peritoneal cavity during draining and filling phases of the PD cycle. The extended TPM theory includes an additional compartment for fill-and-drain phases and combines osmotic water transport, small and middle molecules clearance and adsorption of glucose [141].

Starting from the filling phase, the net volume flow though the peritoneal membrane during APD is given by the sum of six terms [141]:

(43) $$\frac{dV_{PD}}{dt}=J_{v,A}+J_{v,S}+J_{v,L}-L+J_{fill}-J_{drain}$$

Three first terms in the Equation (43) are the water net flow across aquaporins (index “A”), the “small pores” (index “S”) considered as highly selective pathways and “large pores” (index “L”), as weakly selective pathways, respectively. To include the fill (inlet) and drain (outlet) phases, two volume flows have been added. Finally, the flow L represents the net lymphatic clearance from the peritoneum to the circulation (see, for the first estimation of lymphatic clearance, in [142,143]).

In the TPM, the solute flows for each pathway have been calculated by using the equations [141]:

(44) $$J_{solute,S}^{i}=J_{v,S}\left(1-\sigma_{S}^{i}\right)\frac{C_{P}^{i}-C_{D}^{i}\cdot \exp\left(-Pe_{S}^{i}\right)}{1-\exp\left(-Pe_{S}^{i}\right)}$$

(45) $$J_{solute,L}^{i}=J_{v,L}\left(1-\sigma_{L}^{i}\right)\frac{C_{P}^{i}-C_{D}^{i}\cdot \exp\left(-Pe_{L}^{i}\right)}{1-\exp\left(-Pe_{L}^{i}\right)}$$

where $C_{D}^{i}$ is the concentration for each solute i in the intraperitoneal solution, and $C_{P}^{i}$ is the plasma concentration of solute i, respectively. The notations $Pe_{S,L}^{i}$ are used for the Péclet numbers for the small and large pores, respectively.

The kinetic equations describing the time changes in solute concentration, $\frac{dC_{D}^{i}}{dt}$ (for each $i_{th}$ solute), are given by the formula [141]:

(46) $$\frac{dC_{D}^{i}}{dt}=\frac{1}{V_{PD}}\left\{\left(J_{solute,S}^{i}+J_{solute,L}^{i}\right)-C_{D}^{i}\left(J_{v,A}+J_{v,S}+J_{v,L}+J_{fill}\right)+C_{B}^{i}J_{fill}\right\}$$

and the kinetic equations for the solutes concentration change, $\frac{dC_{B}^{i}}{dt}$, in the drain reservoir (index “B”) are [141]:

(47) $$\frac{dC_{B}^{i}}{dt}+C_{B}^{i}\cdot \frac{dV_{B}}{dt}=\frac{1}{V_{B}}\left\{C_{D}^{i}\cdot J_{drain}-C_{B}^{i}\cdot J_{fill}\right\}$$

The reservoir volume $V_{B}$ kinetics is described by the difference in draining and filling flows:

(48) $$\frac{dV_{B}}{dt}=J_{drain}-J_{fill}$$

Within the thermodynamics of membrane transport, volume flows in Equations (43)–(48) can be written as follows [141]:

(49) $$J_{v,A}=\alpha_{A}L_{P}S\left\{∆P-RT\sum_{i=1}^{N}\phi_{i}\left(C_{D}^{i}\left(0\right)-C_{D}^{i}\right)\right\}$$

(50) $$J_{v,S}=\alpha_{S}L_{P}S\left\{∆P-RT\sum_{i=1}^{N}\phi_{i}\sigma_{S}^{i}\left(C_{D}^{i}\left(0\right)-C_{D}^{i}\right)\right\}$$

(51) $$J_{v,L}=\alpha_{L}L_{P}S(∆P-RT\sum_{i=1}^{N}\phi_{i}\sigma_{L}^{i}\left(C_{D}^{i}\left(0\right)-C_{D}^{i}\right)$$

where $\alpha_{A,S,L}$ represent fractional hydraulic conductances for the different water pathways, and $\phi_{i}$ give the osmotic coefficients for each of solute i. The reflection coefficients $\sigma_{S,L}$ are considered to be the same for the solute transport and osmotic transport [32].

By solving Equations (49)–(51) together with Equations (44)–(48) and by applying appropriate initial conditions, Öberg and Rippe obtained $V_{PD}\left(t\right),\;V_{B}\left(t\right),\;C_{D}^{i}\left(t\right),\;C_{B}^{i}\left(t\right)$ functions, respectively, and then, applied the results for the osmotic water transport parameters (such as ultrafiltration, UF) and molecules (urea) clearance calculations for the different dialysate flow rates (DFR) [141]. Numerical calculations based on the extended TMP model were performed for N=25 number of APD cycles for various DFR and different glucose concentrations [141]. The simulations of APD cycles for three different PD regimes, slow, fast and average, allowed researchers to evaluate the optimal dialysate flow rates both for UF and small/middle molecules clearance.

### 4.5. Size-Selectivity of Synthetic Dialysis Membranes: Log-Normal Distributed + Shunt Model

In addition to the two-pore model, as discussed in Section 3.3, a heteroporous model with a log-normal distributed population of pores in parallel with a non-selective shunt (corresponding to the transport across large pores) was used to validate the experimental filtration data [51]. Within the so-called fractional clearance model (or θ–model), the theoretical θ data were calculated from the non-linear convection/diffusion equation combined with a non-selective shunt. Under these assumptions, the θ-model yields [51]:

(52) $$\theta_{model}=f_{D}\frac{1-\sigma }{1-\sigma e^{-Pe}}+f_{L},$$

where the Péclet number is

(53) $$P_{e}=f_{D}\frac{GFR\left(1-\sigma \right)}{P\cdot S}.$$

and $f_{L}=1-f_{D}$ denotes the fractional fluid flow through shunts.

To evaluate the size-selectivity of membranes, the theoretical θ values obtained in the model were compared with the experimental data for FITC-Ficoll filtration of different synthetic dializing membranes and to that of the rat glomerular filtration barrier [51].

#### New Developments

Going in this direction, a new development includes the effects of membrane fouling. Current work of Polyakov and Zydney [144] combines a complete blocking model (see, for the classical complete blocking theory, papers [144,145,146]) and the approach based on thermodynamics of membrane transport. In the framework of thermodynamic description extended to the case of diffusion across the fouling layer, the volume flux can be written as follows:

(54) $$J=\frac{∆P-\sigma ∆\Pi }{r_{m}+r_{F}}$$

where $r_{m}$ and $r_{F}$ are the hydraulic resistance of the membrane and of the fouling (cake) layer, respectively [144]. Based on formulation developed in [144,147,148,149], model calculations for two pores sizes and permeate fluxes as well as the calculation of solute rejection coefficients were carried out in [144,149].

Recent publications on modeling of UF membranes performance based on similar theoretical framework have been reviewed in [150,151,152,153]. In particular, the effect of pore size distribution on permeability of membranes has been studied in detail in [149,150,151,152]. The influence of pore size distribution and pore connectivity distribution has been studied on the diffusive transport in model porous networks by Armatas and co-authors [153]. The results of calculations proved that the pore size distribution and related percolation phenomena essentially affected the tortuosity and diffusivity of the porous network [154].

Modern polymer technologies allow fabrication of synthetic membranes with predetermined geometry of pores (see, for the review, [151,152,153,154,155,156,157,158,159,160,161]). Silicon membranes are convenient objects for the analysis of the effects of pore size and pore geometry. In their recent study Kanani, Fissel and co-authors [152] compared the permeability of silicon membranes with pores of slit-shaped and cylindrical geometry. Model calculations have shown that the membranes with slit pores demonstrated better performance (i.e., showed higher selectivity at given permeability) than ones with cylindrical pores [152]. The effect of this “improved performance”, however, was found to decrease with the increase pore size distribution [152]. Mathematical modeling of transport across tortuous polymer membranes [144,151,162,163] provide new important insights into the process of ultrafiltration. These results demonstrate the complex interrelation between the effects of pore geometry and size distribution.

**Figure 3:**
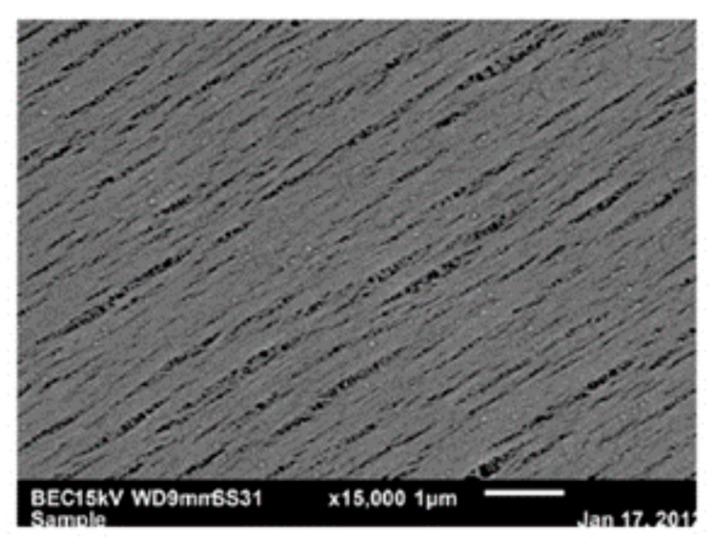
Electron microscopy of inner (blood-contacting) surface of the Polyflux polymer filter membrane at 15,000× showing the slit pores morphology (adapted from [160] with permission).

The slit-shape geometry of the pores on inner surface of the polymer filter membrane is shown in Figure 3.

## 5. Water Transport across Artificial Dialysis Membranes

**Figure 4:**
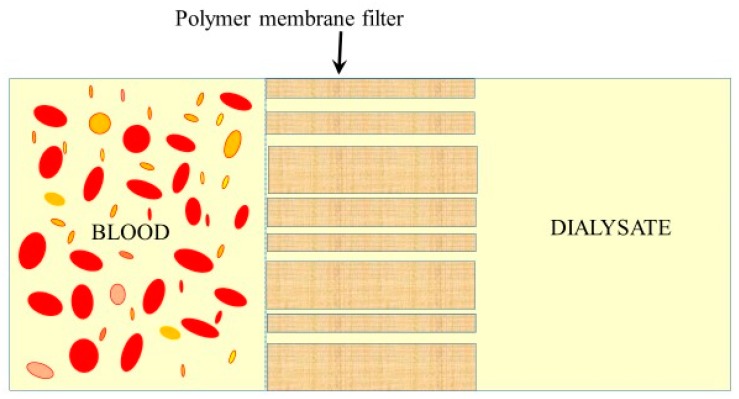
Schematics of the hemodialysis filtration.

Artificial kidney substitutes the natural renal function with the process of blood purification by the hemodialysis procedure using filter membranes of various chemical composition and microstructure (for the review, see [152]). Figure 4 illustrates the schematics of membrane filtration during hemodialysis.

Since HD blood purification demands multidisciplinary efforts, both improvements in engineering and continued search for the optimal filters in biomedicine need to proceed along with clinical studies of new synthetic materials. The main goal of the improved filtration in hemodialysis (HD) is to designing artificial polymer membranes for dialysis capillary filters that have special properties [155,156,157,158,159,160]. This general perspective involves several aspects. First, the technological aspects require polymers with special physical and chemical properties. Second, a design of better HD filters requires new mathematical methods of modeling and computer simulations to study and analyze pore size and geometry, including topological aspects, connectivity of pores and tortuosity factor.

There are special requirements for membranes that are used in hemodialysis, such as adequate diffusion and convection characteristics, sieving coefficients, a cut-off point similar to that of glomerulus [155,156], adequate ultrafiltration rate, suitable biocompatibility properties, non-toxicity of the material, as well as the non-degradable stability under sterilization (which may affect physical properties of the filters) and low-cost solutions [155,161]. In addition, reproducibility of dialysis and constant performance during the entire procedure is a prerequisite of HD [155,161].

Biocompatibility of polymer membranes is an important concern in HD applications [164,165,166,167,168,169,170,171,172,173,174,175]. Biophysics and biochemistry of the membrane biocompatibility (blood compatibility) among the most difficult areas of work, with basic mechanism still waiting to be uncovered.

### 5.1. Microporous Membranes for Hemodialysis: Ultrastructure of Pores and Their Characteristics

**Figure 5:**
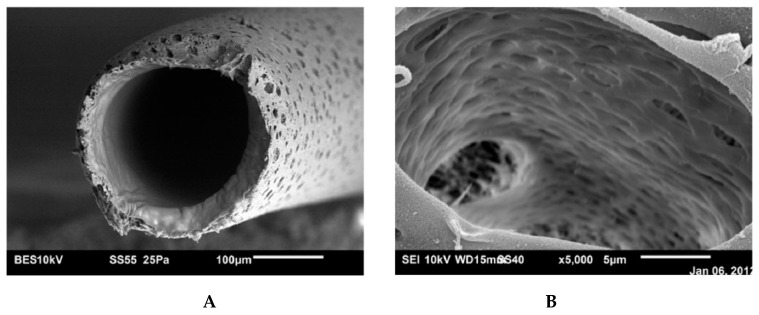
(**A**) SEM of the cross section of a Polyflux capillary at 250×; and (**B**) SEM of a Polyflux capillary wall showing tortuous pore morphology (outside pore in contact with the dialysate) at 5000× (adapted from [160] with permission).

**Figure 6:**
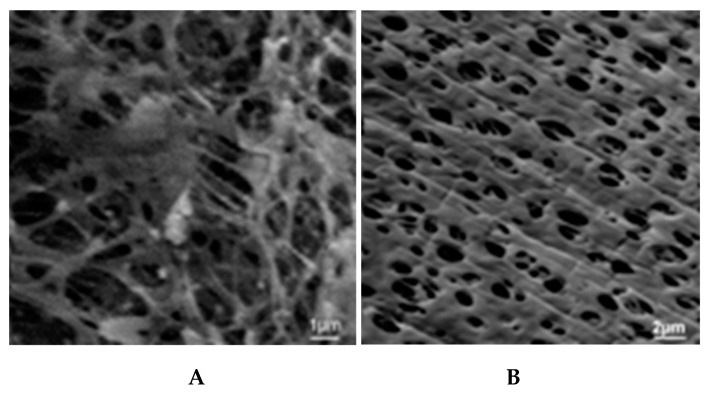
EM of the polysulfone (PSf) capillary ultrastructure: (**A**) EM of the sublayer voids (a sponge-like membrane area) in the dissected capillary wall; and (**B**) EM of the outer surface of PSf capillary (Electron microscopy: REMMA-101A, AO “SELMI”, accelerating voltage 20 kV).

Despite variations in their chemical composition (common polymer materials for hemodialysis membranes are polysulfone (PSf), polyethersulfone (PES), polyamide (PA) and cellulose acetate (CA) [155,160,161]) and microscopic details of the pores, hollow polymer capillaries used in UF share a number of common structural features. In the hemodialysis filter capillaries, the inner surface of a capillary is a dense layer of polymer with nanometer pores (blood contacting surface, Figure 5A). This inner layer borders a highly porous sublayer, a “spongy” material of large voids separated by polymer fibers (Figure 6A). The outer surface of the layered capillary wall is in contact with dialysate (Figure 5B and Figure 6B). The structure of this layer is different from the skin layer and the porous sublayer.

Characterization of polymer membranes used for dialysis includes different techniques [157,159]. Electron microscopy of capillary wall of HD filters is a convenient tool for morphological characterization of membrane’s structure. The structure varies from layer to layer. Numerous research papers reported the ultrastructure of the inner “sponge” membrane area for different type of polymers [157,158,159,160,161,162,163].

### 5.2. Fouling of Dialysis Filters

**Figure 7:**
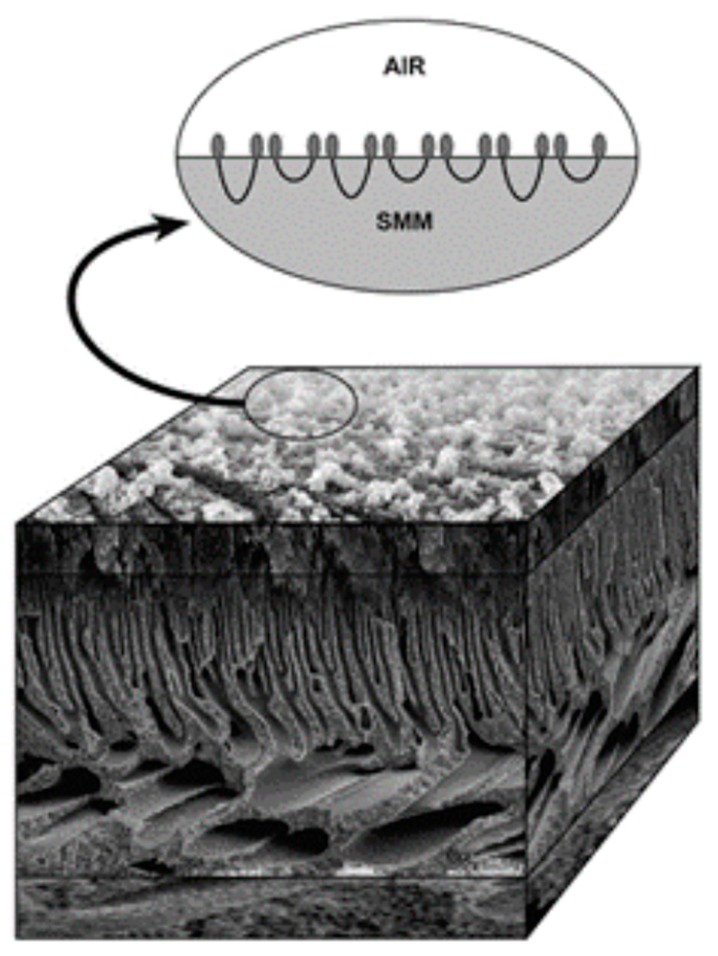
Ultrastructure of filter membrane’s skin layer after its surface functionalization with chemical groups (adapted from [172], with permission).

Current research in the material science of membrane filters for dialysis includes essential efforts in prevention of the biofouling and cake layers formation [163,164,165,166,167,168]. Search for new blended polymers [169] is a rapidly developed branch in physics and chemistry of polymers. Another direction is a surface functionalization of dialysis filters with macromolecular compounds possessing antithrombotic properties and polymers reducing membranes fouling [170,171,172]. Surface functionalization of the membranes may essentially change the architecture of their skin layer (Figure 7). An experimental example of surface functionalization is provided by grafted polymer coating of dialysis filters [172].

Due to the competitive adsorption of high molecular weight proteins (such as albumin, fibrin, fibronectin or globulins, as well as the adsorption of low and medium molecular weight proteins such as beta2-microglobulin, cytokines), the formation of a cake ad-layer occurs, creating a barrier for the transport of fluid. The structure of the formed cake layer—random or ordered—determines the pores geometry (straight channels, randomly distributed voids or tortuous pathways), which may influence the transport of fluid across the cake layer.

Special attention has been paid to heparin, which is widely used as an antithrombotic substance in dialysis circuits [173,174,175]. Permeability of dialysis membranes for water and solutes has been experimentally studied for pristine as well as surface-functionalized filters. In particular, for the heparin coating (of cuprophan membranes), enhanced water permeability has been discovered (in treated versus untreated membranes) [173].

Theoretical consideration of fouling and ultrafiltration process in the cake layers complements quantification of complex dialysis processes. The next section represents a short review of theoretical models describing hindered diffusion through synthetic membranes of composite structure.

## 6. Mathematical Modeling of Molecular Transport in Tortuous Dialysis Membranes

### 6.1. Modeling Diffusion across the Tortuous Membrane Pathways

#### 6.1.1. The “Hydraulic Tortuosity” Notion

**Figure 8:**
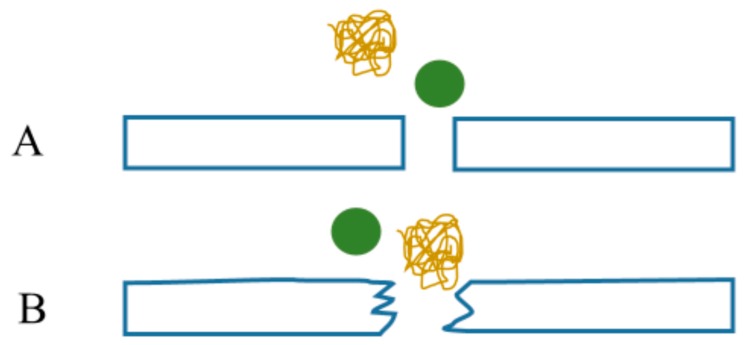
Schematic depiction of the membrane pore: (**A**) cylinder channel pore; and (**B**) tortuous pore.

The complexity of the membrane ultrastructure visualized electron microscopy, possess a challenging task to include in the model not only geometrical size of pores but also the topology of the porous space. The microscopic pores topology has been suggested to characterize in terms of interconnectedness of their shapes, such as “connectivity” and “tortuosity factor” [176]. Notion “tortuosity” it is customary to apply to the analysis of fluid flows across granular beds [177,178,179] or porous media [153,154,162,176]. The sketch of the tortuous membrane pore is shown in Figure 8.

Tortuosity factor was introduced in the fundamental work of Carman [178] as a square ratio of the effective average path length, $L_{eff}$, to the shortest distance, L, along the fluid flow direction [176]:

(55) $$\tau ={\left(\frac{L_{eff}}{L}\right)}^{2}$$

In the later models, instead of “tortuosity”, the notion of “hydraulic tortuosity” of porous liquid-filled media has been suggested [176]. The “hydraulic tortuosity” factor was introduced as the ratio of the cross section available for conduction (flow) to the bulk cross section which was assumed to be equal to the bulk (or volume) porosity ε. The total “pore volume”, V, can be defined as $V=\varepsilon L^{3}$, respectively [176]. In the next section, the derivation of Equation (55) is provided by using the analogy between the electrical conduction and diffusion in porous media (see Section 6.1.2 and Section 6.1.3, and “Formation resistivity” in particular).

In various models, volume porosity value ε has been related to the fluid flow characteristics. For example, the expression for the averaged velocity in the porous media flow channels (or “pore velocity”) in Hagen–Poiseuille type equation is given by the formula [176]:

(56) $$v_{inside-the-pore}=\frac{∆P}{L_{eff}}\cdot \frac{D_{h}^{2}}{16K\eta }$$

where ΔP is the hydrostatic pressure, $D_{h}$ is the “hydraulic diameter”, K is the “shape factor”, η is the shear viscosity of fluid. The hydraulic diameter was shown can be related to the specific surface area of solid volume, $A_{solid}$, and volume porosity ε as follows [176]:

(57) $$D_{h}=\frac{4\varepsilon }{A_{solid}\left(1-\varepsilon \right)}$$

The usual form of the Carman-Kozeny expression for the permeability coefficient $k\equiv k_{CK}$ is given by the relation [176]:

(58) $$k_{CK}=\frac{\varepsilon^{3}}{K\tau^{2}{\left(1-\varepsilon \right)}^{2}A_{solid}^{2}}$$

#### 6.1.2. Analogy between Electrical Conduction and Diffusion in Porous Media

One of the most useful physical parallels for the analysis of mass transfer in porous materials is the analogy between diffusion in porous media and electrical conductivity [176,179].

The conductivity of a porous medium (proportional to the porosity) is often described by using a resistivity (or formation factor, Φ) introduced as an inverse to ε value. It was, however, discovered that Φ(ε) has a more complicate form [176]. In particular, by using the analogy with the electrical characteristics of non-conducting (solid phase) particles embedded into conductive medium (fluid inside the pores), one can use the expression for the conductivity (or resistivity) of the mixture. For instance, for the uniform non-conductive spheres in bulk fluid, the Maxwell model [180] provides the following expression which relates the resistivity (formation factor) Φ to the volume porosity ε:

(59) Φ=(3−ε)/2ε.

The electrical-fluid flow analogy and Maxwell’s expression (Equation (59)) have been used in many studies of flows through granular materials, in particular, for the analysis of transport in tortuous solid (colloidal) and soft (biofilm) cake layers (for the review, see [162]).

The next section shows how, in a rather simple scheme, the formation factor Φ and porosity ε can be related to the geometrical parameters of a porous medium or its tortuosity factor.

An important physical clarification about this parallel to electrical conductivity and diffusion should be made. For a consistent view, let us consider conductivity of an anisotropic material [181]. Generally, the relation between the electrical current density j and the electrical field E is:

(60) $$j_{i}=\gamma_{ik}E_{k}$$

where $\gamma_{ik}$ is a symmetric tensor of conductivity. The symmetry of conductivity tensor

(61) $$\gamma_{ik}=\gamma_{ki}$$

follows from Onsager’s principle of symmetry of kinetic coefficients.

By introducing dynamic variables describing the state of the system at each point $\left\{\chi_{n}\right\}$, the velocities $\frac{\partial \chi_{n}}{\partial t}$ and corresponding generalized forces, $X_{n},$ one can write for the time variation of total entropy Θ of the system [181]:

(62) $$\frac{d\Theta }{dt}=-\int \sum_{n}X_{n}\frac{\partial \chi_{n}}{\partial t}dV$$

In particular, for the time variation of the entropy of the anisotropic conductive body in electric field, when a current flows in a conductor [181], one can get:

(63) $$\frac{d\Theta }{dt}=\frac{1}{T}\int \left(\vec{j}\cdot \vec{E}\right)dV.$$

where T is temperature and V is the body volume.

By comparing Equations (61)–(63), one can easily establish the correspondence between the components of the electrical current density and velocities [181]:

(64) $$\frac{\partial \chi_{n}}{\partial t}\to j_{n}$$

and between the generalized forces and components of the electric field

(65) $$X_{n}\to -\frac{E_{n}}{T}.$$

From the above-mentioned theoretical consideration one can see, that linear equations describing the electrical conduction and diffusion in liquid phase have a similar structure and obey Onsager’s principle of symmetry of kinetic coefficients. This similarity has a fundamental consequence considered in the Section 6.1.3. The electrical conduction–diffusion analogy has been extensively used in the theoretical analysis of molecular transport through porous media and granular materials [162,176,179].

#### 6.1.3. Formation Resistivity Factor and Tortuosity

The kinetic equations describing diffusion in a liquid and electrical conduction are linear relations between the thermodynamic fluxes and conjugated forces, respectively. In an open space filled with liquid, these equations are written in the form of Fick’s law and Ohm’s law for the mass flow $j_{m}$ and current density, $j_{e}$:

(66) $$j_{m}=-D\nabla n,$$

(67) $$j_{e}=-\gamma \nabla U,$$

where D and γ are the diffusion coefficient and the specific electrical conductance, respectively, ∇U is the electrical potential gradient.

Diffusion of solutes (for example, trace molecules) through a fouling (cake) layer can be described in terms of hindered diffusion in a porous medium. For the hindered diffusion of solutes in a porous fouling layer of porosity ε, one can rewrite Equation (66) as follows [162]:

(68) $$j_{H}=-\frac{D_{0}}{\Phi }\nabla n=--D_{H}\varepsilon \nabla n$$

where Φ is the formation factor, $D_{H}$ is the hindered diffusion coefficient of solutes in the fouling (cake) layer. Tortuosity factor $\tau =\frac{D_{0}}{D_{H}}$ is the ratio of the diffusion coefficients, respectively [162].

In a fouling (cake) layer filled with liquid, solute molecules diffuse across conducting capillaries in a solid matrix. Here, the effective diffusion coefficient and electrical conductivity become a function of two factors that characterize the medium: its porosity (ε) and the shape of the conductive capillary (pore) or tortuosity. According to [176], tortuosity of porous materials is a fundamental property which measures the deviation from the macroscopic flow at every point of fluid. In general, tortuosity is a tensor value, yet for isotropic material the tortuosity tensor reduces to a scalar (tortuosity coefficient, τ) [176].

The relation between tortuosity of the fouling layer, its porosity and the resistivity formation factor can be clarified by using the following example.

**Figure 9:**
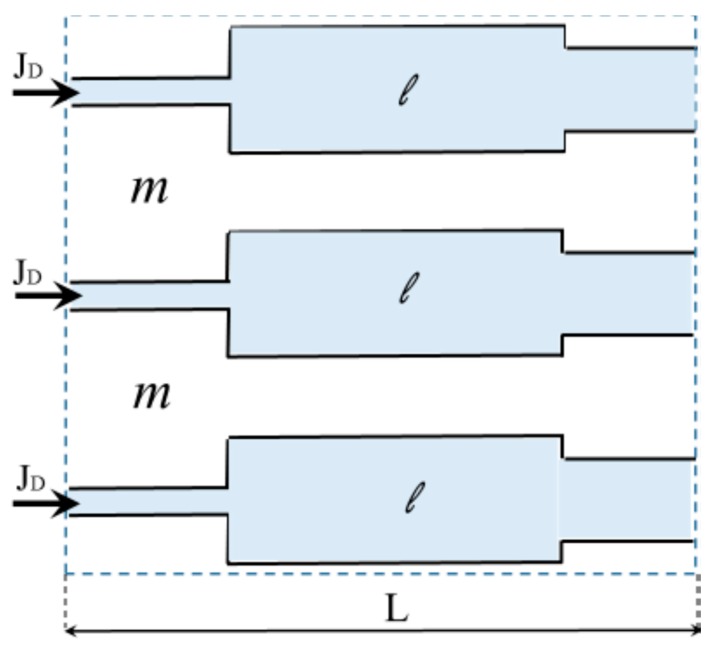
A sketch of porous material represented as a composite of solid matrix (index “m”) and tortuous channels filled with liquid (index “l”).

Porous medium geometry in its simplest form can be represented as a uniform subset of conductive capillaries (channels in a solid matrix) having the same length but varying in diameters (Figure 9) [176].

**Figure 10:**
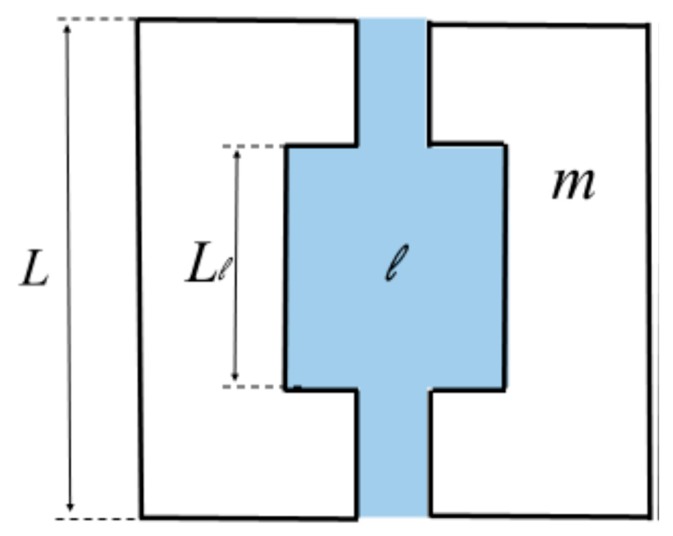
Actual pore geometry of length L in the uniform capillary model of [176].

For the porous material depicted in Figure 10, the tortuosity τ is given by the geometric ratio:

(69) $$\tau ={\left(\frac{L_{l}}{L}\right)}^{2}$$

and the material’s porosity ε (which is the ratio of the void space and solid matrix volume) is expressed as follows [176]:

(70) $$\varepsilon =\frac{L_{l}a_{l}+L_{m}a_{m}}{A\left(L_{l}+L_{m}\right)}$$

Besides porosity and tortuosity, another parameter called the resistivity formation factor, Φ, is equally widely used. According to [176], the resistivity formation factor can be introduced as a ratio of the electrical resistance $R_{p}$ of a porous material filled with ionic solution, to the bulk resistance $R_{l}$ of this solution in a volume occupied by porous space, $\Phi =R_{p}/R_{l}$. This value provides a measure to evaluate the influence of porosity on the electrical resistance of material. In the model approach, the resistivity formation factor and porosity were related as following [176]:

(71) $$\Phi \equiv \frac{\Upsilon }{\varepsilon }$$

where Υ is “electrical tortuosity” given by the ratio of molecular diffusivity to the effective molecular diffusivity [176]. For the porous sample presented in Figure 9, the resistivity formation factor Φ is given by the ratio:

(72) $$\Phi =\frac{R_{p}}{R_{l}}=\frac{\rho_{l}\left(L_{m}/a_{m}+L_{l}/a_{l}\right)A}{\varrho_{l}\left(L_{m}+L_{l}\right)A}$$

By using Equation (71) and the relation $L=L_{m}+L_{l}$, one can find from Equation (72) that

(73) $$\Phi =\frac{1}{\varepsilon }{\left(\frac{L_{l}}{L}\right)}^{2}=\frac{\tau }{\varepsilon }$$

Comparing (69) and (73), one can notice the equivalence of these expressions, so for the geometry of the pore presented in Figure 10, Υ=τ.

For a more complex geometry of the channels, a generalized expression for the resistivity formation factor was introduced:

(74) $$\Phi \equiv \frac{\tilde{\Upsilon }}{\varepsilon },\;\tilde{\Upsilon }=\tau \cdot \tilde{S}$$

where $\tilde{S}$ is the ‘constriction factor’ ($\tilde{S}\geq 1)$ [176].

### 6.2. Geometrical Model of Hindered Diffusion in Fouling Layer Composed of Microspheres

**Figure 11:**
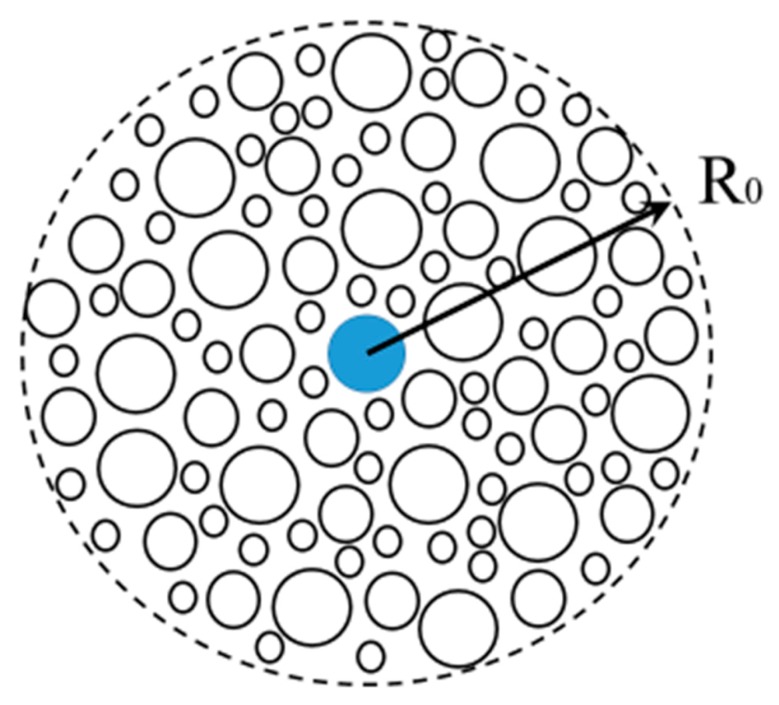
A sketch of homogeneous and isotropic swarm of microspheres of porosity ε illustrating the geometrical model [182]. The figure shows cross sections running through the center of a reference microsphere (marked with blue) of radius $R_{1}$ and an outer sphere with radius $R_{0}$ (a concentric shell, marked with a dotted line).

Neale and Nader [182] formulated a geometrical model which provides a deep physical insight into diffusion processes in fouling layers. The authors analyzed the transport in a porous medium, a spherical cavity in a homogeneous isotropic swarm composed of microspheres of different size (Figure 11).

In the geometrical model suggested in [182], the ratio of radii:

(75) $$\frac{R_{0}}{R_{1}}={\left(1-\varepsilon \right)}^{-\frac{1}{3}}$$

where ε is the porosity of the material that introduced as a ratio of outer shell volume to the reference volume of the spheres:

(76) $$\frac{v_{0}}{v_{1}}=1-\varepsilon .$$

By solving kinetic transport equations

(77) $$j_{10}=-D_{1}\nabla n_{1}$$

(78) $$j_{0\infty }=-D_{0}\nabla n_{0}$$

with the appropriate boundary conditions for the spherical shell ($R_{1}<R<R_{0}$) and the exterior porous material ($R_{0}<R<\infty$), the authors calculate corresponding macroscopic fluxes $j_{10},j_{0\infty }$ and evaluate the diffusivity factor $\Lambda =\frac{D_{0}}{D_{1}}$ (defined as a ratio of effective diffusivity in the porous medium to the absolute diffusivity, i.e., diffusion in the fluid without obstacles):

(79) $$\Lambda =\frac{2\varepsilon }{3-\varepsilon }\;(0\leq \varepsilon <1)$$

In fact, the diffusivity factor Λ used by the authors is the inverse formation factor Φ, so the result in Equation (79) is reduced to Maxwell’s formula (Equation (59)) [182]. The authors emphasize that the boundary value electrical conduction of mixture [180,183,184] and the diffusion problem solved in the proposed geometrical model are mathematically equivalent.

### 6.3. Diffusive Tortuosity and Random Walks Simulations

Membrane fouling is one of the major problems in filtration [144,148,162,167]. The result of fouling due to solute adsorption onto the membrane surface (with proteins topping the foulant list) is the gradual flux decline during the filtration. The interaction between the dialyzing membrane and proteins as well as membrane hydrophilicity and porosity are the crucial physico-chemical factors in fouling. The impact of porosity and pore sizes on the fouling mechanism have been studied in a number of theoretical works [144,145,146,147,148,149,150,151,152,153,154,162]. It was shown, that molecules smaller or of diameter comparable to that of the pores can penetrate the pore and reduce its effective size (or completely block it). This mechanism is called adsorptive fouling (or pore blocking mechanism, respectively) [144,145,146]. In the case, when the solute molecules’ size exceeds that of the pore opening, they tend to form a cake layer deposited onto the membrane surface. Various scenarios of membrane fouling—from standard and intermediate blocking to complete pore blocking and formation of cake layers—have been considered in [144,145]. Physical mechanisms of pore blocking due to solute adsorption have been analyzed in [144]. Several special models (“classical standard blocking”, “m-Model” and “depth filtration model”) and different pore foulant profiles inside the pore have also been suggested [144].

In the search for the new and improved membrane filter materials and best modes of dialysis procedure, one should thus consider hindered diffusion in cake layers. Despite its unavoidable simplifications of real pores’ structure, theoretical modeling provides significantly better understanding of the diffusion mechanism in tortuous pathways.

Diffusion tortuosity factor of solid and soft fouling (cake) layers has been investigated in current theoretical work [162]. In this publication, random walk simulations of solute traces were carried out for different geometries of the porous cake layer for (for both periodic and random pathways). Within the hindered solute diffusion theory, the authors have modeled fouling and ultrafiltration in colloidal porous cake layers [162]. In particular, by using Maxwell’s formula (Equation (59)), the authors have related the diffusive tortuosity factor to the cake volume fraction and calculated corresponding diffusive flows [162].

Theoretical modeling of hindered diffusion is an extensive area of research which cannot be completely covered in one short review. The basic models providing valuable insight into the physical mechanisms of hindered molecular transport and pore blocking can be found, for example, in [32,144,145,146,147,151,152,153,154,162,185]. Recent developments in mathematical modeling include analysis of diffusion across asymmetric structures of tortuous layers on the surface of membrane filters [162] and other porous media with complex structure [185,186]. The authors of publication [185] have suggested a theoretical model for the diffusion in porous media with periodic microstructure and introduced a time-dependent diffusion coefficient, D(t). Its calculated effective D(t) value also proved to be dependent on the absolute size and topology of the porous microstructure. The authors calculate the effective diffusion coefficient by using the analogy of stationary diffusion and electrical conduction in the pore space [185].

## 7. Theoretical Modeling of Red Blood Cell–Polymeric Membrane Interaction

The interaction of blood cells with artificial surfaces is an issue crucial for dialysis. Adhesion of blood components adhesion to the dialysis membrane filters may lead to unfavorable effects such as clotting and thrombosis.

The reaction of red blood cells (RBCs) to the polymeric surfaces is a factor of prime importance in the search for biocompatible blood-contacting materials. The complex processes involved in cell–surface interaction result in different morphology of RBCs which can be observed microscopically [187,188,189,190,191,192,193].

### 7.1. Cell Shape Modeling

The shape of a RBC represents a mechanical response of its three-layer membrane (composed of an outer glycocalix layer, a lipid bilayer with proteins, and an underlying cytoskeleton) to various physico-chemical stimuli, i.e., changes in solute concentrations, temperature variations, mechanical stress, addition of pharmacological compounds or contact with artificial surfaces. In hypotonic media, normal discoid shapes of RBCs are easily transformed into spheres. The crenated forms or echinocytes are observed in hypertonic solutions [187]. When all but one factor that can potentially influence the cell shape are controlled, this factor (for example, a contact with an adhesive surface) can be considered as the main reason behind the visible morphological transformations. RBC geometry can thus be considered to be the sensor cell’s response to contact with a foreign surface [191,192,193].

Closed lipid bilayer (vesicles) are convenient models for studying mechanical properties of RBCs. The softness of lipid bilayer to bending is a reason for microscopically observed conformations of vesicles [188,189,190,191,192,193,194,195,196,197,198,199,200,201,202,203,204,205,206]. The similarity between these conformations and some of the RBC shapes has been the subject of extensive theoretical research since the 1970s [187,188,189,190,191,192,193,194,195,196,197,198,199,200,201]. The most universal and successful approach, the curvature elasticity theory [198] pioneered in Helfrich, Deuling and Canham works [194,195,196,197,198], is briefly reviewed in the next subsection. Since the initial publication of Helfrich’s ansatz of curvature elasticity [198], rapid progress in the analysis of the geometry of lipid vesicles and RBC has been achieved [200,201,202,203,204,205,206,207,208,209,210,211,212,213,214,215,216,217,218,219,220,221,222,223,224,225,226,227,228,229,230].

#### 7.1.1. Helfrich’s Curvature Elasticity Theory

Helfrich (or Canham–Helfrich) theoretical model of curvature elasticity combines continuum mechanics of thin elastic shells with the differential geometry approach. The model allows researchers to calculate the equilibrium shapes of closed membrane shells considered as elastic surfaces. According to the curvature elasticity anzats, the equilibrium shape of the closed membrane surface can be found by minimizing its free elastic energy $F_{C}$ which is given by the following formula [198]:

(80) $$F_{C}=\oint (\frac{k}{2}\;{\left(2H-C_{0}\right)}^{2}+\bar{k}K)dA,$$

(81) $$H=\frac{C_{1}+C_{2}}{2}$$

(82) $$K=C_{1}C_{2}$$

where H and K are the mean curvature and Gaussian curvature of the membrane surface, respectively, A denotes the surface area of membrane. The elasticity of the membrane is described by bending elasticity, k, and splay elasticity, $\bar{k}$ moduli that can be experimentally measured (the standard values for the moduli are $\sim 10^{-19}J$) [195,196,197,198,218]. The first term in the expression (80) is also known as Willmore functional. Model parameter $C_{0}$, the spontaneous curvature of the membrane, has been intensively debated subject since Helfrich [198] introduced it as a curvature of a closed membrane for the absolute minimum of the free elastic energy (80) (i.e., corresponding to the membrane “ground state”). The alternative explanation for some experimental RBC shapes (for example, echinocytes) is based on a “bilayer couple” model [199,200,222].

To account for the deformations arising due to the changes in the osmotic pressure, ∆p, and/or membrane surface tension, σ, the elastic energy (Equation (80)) should be re-written as follows [195,196,197,198]:

(83) $$F=F_{C}+\Delta pV+\sigma A.$$

The equilibrium shape of lipid vesicles can be found from the variation of the elastic free energy:

(84) $$\delta \left(\frac{k}{2}\int {\left(C_{1}+C_{2}-C_{0}\right)}^{2}dA+\Delta pV+\sigma A\right)=0.$$

Corresponding Euler-Lagrange equation for the variational problem (Equation (84)) together with the conditions for the parameters Δp and σ (Lagrange multipliers), has been analyzed in the pioneer studies by Deuling and Helfrich [195,196,197,198]. The analysis resulted in the calculated catalog of vesicle shapes [195].

#### 7.1.2. Contact Potential

Variational analysis based on the curvature elasticity theory and Helfrich’s anzats (Equations (80)–(84)) allows researchers to calculate the equilibrium shape of free vesicles in solution. Interaction of cell membranes with artificial surfaces was included in the model by incorporating into the free energy an additional term which is proportional to the contact area [205,208]. A detailed theoretical analysis of the interaction of lipid vesicles with an artificial surface plane has been performed in the classical works of Seifert [205] and Sukumaran and Seifert [220], which consider axially symmetric shapes of closed membranes.

Cell adhesion is a complex biological process [211,212,218,219]. To understand the basic physical principles underlying this phenomenon, artificial membranes were designed to mimic and used to mimic and control cell adhesion conditions [211,212,217,218,219,220]. Simplified models considering the cell as a spreading fluid drop, suggest the analogy of cell–surface adhesion and wetting–dewetting transition [211]. The total free energy (work) of adhesion is given by two terms:

(85) $$∆F_{ad}=-WA_{contact}+\sigma ∆A+F_{C}$$

which describe the balance between the gain in adhesion energy (WA) and the cost in bending energy $F_{C}$ and tension-controlled membrane elasticity σ∆A [211].

In a more general form, the free energy of adhesion is the integral of the contact potential W [205,220]:

(86) $$F_{W}=\int_{\Gamma }^{}WdA$$

A calculation of theoretical shapes of red blood cells in contact with artificial surfaces based on Helfrich’s model (Equation (84)) has been published elsewhere [191]. In this work, the membrane is represented as a smooth 2D surface Γ embedded in a 3D Euclidean space. Then, the evolution of the closed membrane surface that evolves from the initial $\Gamma_{0}$ surface is considered under the condition that its area and the enclosed volume stay constant (while its bending energy $F_{C}$ (Equation (80)) decreases). The cells’ equilibrium shapes correspond to surfaces (the stationary points of the Helfrich’s functional in Equation (84)) satisfying the shape equation [191]:

(87) $$2k\left({∆}_{\Gamma }H+2H\left(H^{2}-K\right)\right)+kC_{0}\left(2K-C_{0}H\right)-2\sigma H+\Delta p=0$$

where $\Delta_{\Gamma }$ denotes the Laplace-Beltrami differential operator. For the volume enclosed into the cell and its total surface area be kept constant (if the parameters Δp and σ are considered as time-dependent Lagrange multiplies), the following criteria must be satisfied [191]:

(88) $$\frac{d}{dt}\int_{\Gamma }dA=\int_{\Gamma }2\nu HdA=0$$

(89) $$\frac{d}{dt}\int_{\Gamma }dV=\int_{\Gamma }\nu dA=0$$

If the membrane surface moves with normal velocity ν

(90) $$\nu =-2\left(\Delta_{\Gamma }H+2H\left(H^{2}-K\right)\right)-C_{0}\left(2K-C_{0}H\right)+\frac{1}{k}\left(2\sigma H-\Delta p\right)$$

then, during the evolution of the surface, its Helfrich’s energy $F_{C}$ will not increase. When the evolution of the membrane surface is finished, the final surface will satisfy the shape Equation (87).

Generalizing the approach of Sukumaran and Seifert [220], the membrane–artificial surface interaction has been introduced through addition of term in Equation (86) to the elastic free energy [191]:

(91) $$W\left(m\right)=W_{0}{\left(\frac{\zeta_{0}}{\xi \left(m,\Gamma_{\alpha }\right)}\right)}^{2}\left\{{\left(\frac{\zeta_{0}}{\xi \left(m,\Gamma_{\alpha }\right)}\right)}^{2}-2\right\},\;\xi \left(m,\Gamma_{\alpha }\right)\in \Gamma_{\alpha }$$

In the contact potential expression (Equation (91)), parameter $\zeta_{0}$ denotes the distance from the point where the interaction force is zero, and $\xi \left(m,\Gamma_{\alpha }\right)$ denotes the distance between a point m and the artificial surface, $\Gamma_{\alpha }$. The intensity of adhesion (the adhesion strength) is given by constant $W_{0}$. In this approach, the membrane is weakly attracted to the $\Gamma_{\alpha }$ when $\zeta >\zeta_{0}$ and repelled when $\zeta <\zeta_{0}$.

Calculated cell shapes contacting the planar surface have been obtained for a number of different adhesion intensities. The discocyte-like shapes were found at low adhesion intensity values. Increasing the adhesion parameter $W_{0}$ produced convex shapes and caused the appearance of the highly curved region at the contact perimeter. The results of the modeling have been compared to the experimental observations of RBCs strongly adhered to a plane poly-L-lysine coated glass and weakly adhered to the surface of polysulfone dialysis membrane.

**Figure 12:**
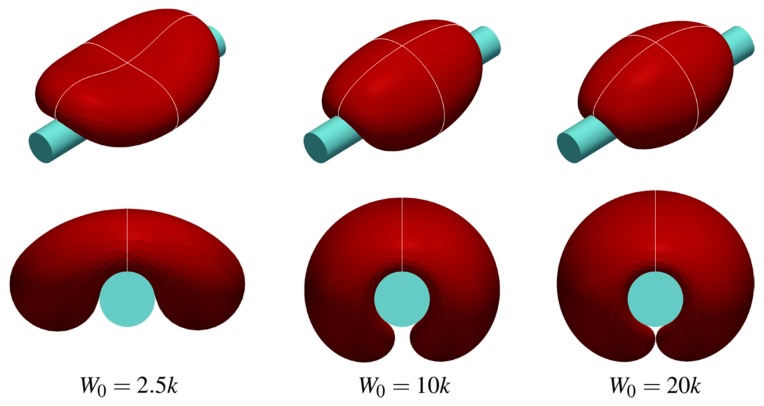
Computed shapes of a RBC in contact with cylinders for different adhesion (adapted from [191] with permission).

**Figure 13:**
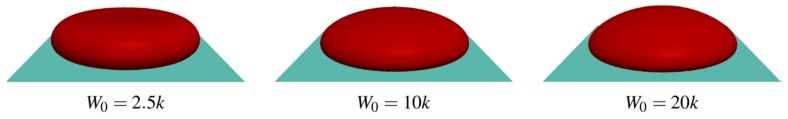
The computed shape of the RBC in contact with a plane for different adhesion intensities (adapted from [191] with permission).

The extended model also allows us to study the effects of artificial surface’s non/planar geometry on cell morphology, in order to calculate, for example, the shape of RBCs contacting a network of cylindrical polymer fibers [191]. In particular, it was shown that under certain conditions, RBCs may adapt their shape to the fiber’s surface at the contact zone. Calculated shapes of RBC contacting artificial surfaces with different properties are shown in Figure 12 and Figure 13.

#### 7.1.3. The Role of Membrane Tension

Curvature elasticity approach in Equations (80)–(84) considers bending elasticity as the leading deformation in cell shape transformations when the membrane tension is negligible. However, for large deformations of membrane surface, free elastic energy should be re-written to include membrane tension:

(92) $$F_{m}=F_{C}+F_{A}$$

(93) $$F_{A}=\frac{\gamma }{2}\int {\left(∆A/A\right)}^{2}dA.$$

The elastic modulus of RBC membrane stretching has been estimated $\gamma \sim 10^{3}\mathrm{mN}/m$ ($\sim 10^{2}\;\mathrm{mN}/m$ for lipid bilayer membranes) [188,189].

Since the lipid bilayers are convenient objects to study mechanical properties of cell membranes, multiple studies of membrane tension have been performed on vesicles [199,200,201,202,203,204,205,206,207,208,209,210,211,212,213,214,215,218,219,220]. In particular, membrane tension plays a key role in osmotic reaction of cells at hypotonic conditions when the shape of RBCs changes from discoidal to spherical. The theory of osmotic lysis of lipid vesicles, pioneered by Kozlov and Markin [213], predicts the rupture of tense membrane and lysis of cell content once membrane tension exceeds the critical value of the membrane stretching. In the framework of Kedem-Katchalsky approach, the kinetic equation describing water flux $J_{V}$ across a semi-permeable membrane can be written as follows:

(94) $$J_{V}=\frac{dV}{dt}=L_{p}S(∆p-∆\pi_{osm)}$$

where $∆\pi_{osm}=RT\Delta C$ is the osmotic pressure due to the difference of solvent concentration outside and inside of the cell, Δp=γ/2R is the Laplace (hydrostatic) pressure, R is the radius of the membrane sphere, S is the surface area of membrane, and V denotes its enclosed volume. During swelling, after a critical surface tension is achieved, a closed membrane sphere can burst with subsequent formation of a hydrophilic pore [213,214].

The theory of osmotic lysis of closed membranes [213] provides essential clues to understanding the interrelations between water transport across the membrane and cell stability under osmotic stress. Recent experimental studies [215,216,217] reveal the importance of this approach for cell membrane studies.

For cells, effective membrane deformation (or apparent membrane tension) should also account for membrane tension due to deformation of the membrane-cytoskeleton attachments (MCA) [217,218,219,223,224,225,226,227,228,229].

#### 7.1.4. Altered RBC Shapes: Mechanics of the RBC Cytoskeleton

Above-mentioned models successfully describe smooth-shaped membrane surfaces. More complex geometries of RBCs, such as non-axisymmetric discocytes, as well as discocyte-stomatocyte and discocyte-echinocytes transformations have been described within the framework of the so-called ADE (area-difference-elasticity) model [200,201,218,222,223,224]. The central point of the ADE model is the assumption that there is a difference in relaxed surface area, $\Delta A_{0}$, between the outer and inner leaflets of the RBC’s bilayer matrix with the distance δ between their neutral surfaces. According to the “bilayer couple” hypothesis [199,200,223,224], this difference is a source of mechanical deformations, additionally contributing to the free elastic energy of membrane:

(95) $$F_{C}=\frac{k}{2}\oint {\left(2H-C_{0}\right)}^{2}dA+\frac{\bar{\kappa }}{2}\frac{\pi }{A\delta^{2}}{\left(\Delta A-{\Delta A}_{0}\right)}^{2}$$

In the extended ADE model [223], the elastic energy of the RBC cytoskeleton $F_{el}$ has been included as a following term:

(96) $$F_{el}=\frac{\gamma_{el}}{2}\int {\left(\lambda_{1}\lambda_{2}-1\right)}^{2}dA_{0}+\frac{\mu }{2}\int \left({\frac{\lambda_{1}}{\lambda }}_{2}+{\frac{\lambda_{2}}{\lambda }}_{1}-2\right)dA_{0}$$

The first term in Equation (96) corresponds to the elastic energy of membrane stretching ($\gamma_{el}\approx 3\;\mu$ for the cytoskeleton [223]), while the second term is associated with the elastic energy of shear deformation of the cytoskeleton (with the shear elastic modulus estimated $\mu \sim 2.5\;10^{-6}\;J/m^{2}$ [187,223,224,225]). The elastic energy of the cytoskeleton bending is neglected.

The analysis of echinocytes, the spiculated forms of RBCs, allows researchers to estimate the characteristic elastic length scale, $L_{el}$, which is a ratio of the cell membrane bending modulus to the cytoskeleton’s shear modulus [223]:

(97) $$L_{el}=\sqrt{\frac{k}{\mu }}.$$

It was shown that this value of $L_{el}\approx$ 0.28 μm sets the characteristic scale of the spicule size and determines the fraction of spicules [223]. An extended model of stomatocyte–discocyte–echinocyte transformations has been elaborated in [223,224,227,228,229]. Confocal microscopy study of echinocyte shapes and their comparison with the calculated shapes has been recently published in [230].

Mechanical properties of RBC membranes, including cytoskeleton deformation, have been theoretically and experimentally studied in pioneering works by Evans and Skalak [189] and later analyzed by Kozlov and Markin [225]. Extensive theoretical analysis with simulations of cytoskeleton network’s elasticity has been published in [227,228]. When the contribution of bending elasticity is dominant, thermal fluctuations of RBC membrane (both in-plane and out-of-plane) must be taken considered [226].

### 7.2. Morphology of RBCs in Contact with Polymer Surfaces of Dialysis Capillaries

**Figure 14:**
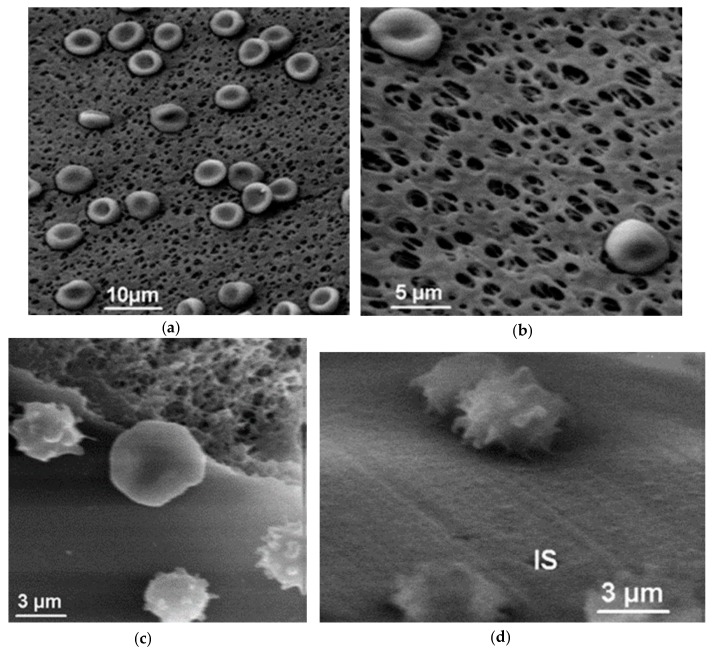
EM of RBCs adsorbed onto the different surfaces of a polysulfone capillary: (**a**) outer surface of the capillary; (**b**) zoomed image; (**c**) inner surface of the capillary (electron microscopy), where the polymer capillary is dissected along the long axis, then flattened and attached to the support; and (**d**) zoomed image of echinocytes on the inner surface (IS) of the capillary. (Electron microscopy: REMMA-101A, AO “SELMI”, accelerating voltage 20 kV. Red blood cells: washed in phosphate buffer pH 7.2, fixation 2% glutaraldehyde (SPI, USA), dehydration in alcohol range 2–98%).

The morphology of RBCs brought into contact with artificial surfaces of various structure and geometry, in particular, with polymer dialysis membranes, has recently been analyzed in [191]. More detailed investigation resulted in observations that RBC shape could be different if adsorbed on the inner or outer surfaces of the capillary wall (Figure 14). Since the chemical structure of the polymer holds for the whole capillary, surface energy of the inner and outer surface can differ due to the variations in the surface density of polymer. This observation of asymmetry in surface properties of polymer capillaries is confirmed by recent experimental work [160], where SEM analysis of both surfaces of the Polyflux hemodialysis filter pores has shown them to have differing ultrastructure (Figure 5). This is clearly manifested on the outer surface of the polymer capillary, as labyrinths of complex passages and voids in the sponge intermediate area under the skin layer, which are, as concluded, not connected.

Experimental results demonstrate the presence of discocytes together with a small number of cup-formed cells visible on the outer surface (Figure 14a,b) and of crenated and echinocyte cell shapes among the cells contacting the inner surface of the dialysis capillary (Figure 14c,d). The occurrence of observed shapes can be explained assuming different surface energy values for inner and outer surfaces of the polysulfone polymer membranes. This observation is in accordance with recent results [231] revealing that surface energy of polysulfone polymer membranes is dependent on the material’s density and degree of porosity (pore size).

**Figure 15:**
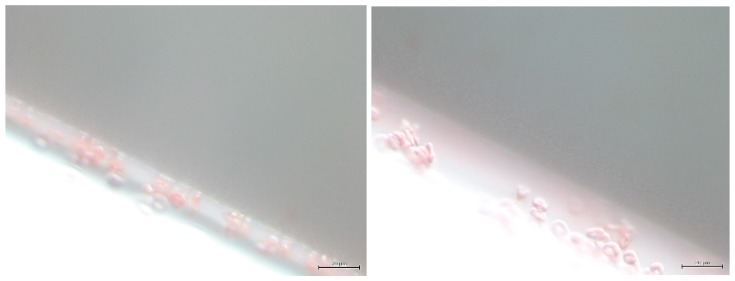
Light microscopy of RBC flow between the glass surface (transparent area) and the outer surface of a polymer (polysulfone) capillary (grayish area). Scale bar 20 μm. Most of the cells retain normal discocyte shapes.

Surface adhesion of blood cells is a highly undesirable process that requires further detailed investigations. One should mention that valuable information on shape transformation of cells in dialysis capillaries as well as other physical characteristics, such as RBCs’ aggregation, deformability and clotting, could be obtained from microfluidic and Lab-on-a-chip experiments [232,233,234,235,236,237,238,239,240]. Recent dynamic studies of RBC shapes in flows reveal that most of the cells retained normal discocyte geometry during the motion between the glass surface and the outer surface of the polymer dialysis capillary (Figure 15).

## 8. Discussion and Outlook

In the current review, we outline major efforts in providing a quantitative description of modeling the water transport processes in dialysis and present key mathematical models that address this issue, while also reviewing briefly the classical models for the membrane transport.

Two common treatment procedures for patients with renal failure are hemodialysis (HD) and peritoneal dialysis (PD), where blood filtration is the goal. In peritoneal dialysis (PD) the filtration occurs in the abdominal cavity across biological semi-permeable peritoneal membrane, while in hemodialysis (HD) (extracorporeal procedure of blood purification from urea, creatinine and excess water) the transport of water and solutes is achieved in dialysis machine via artificial filters made of polymer membranes. Hemodialfiltration (HDF) is another effective renal replacement method which combines diffusion and convection to enhance solute removal in dialysis. The detailed analysis of HDF technical and clinical issues is presented in the current review by Ronco [241].

Patients’ quality of life is the most important concern for the newer designs of dialysis technique [1,242,243,244,245,246,247,248,249,250,251,252,253,254]. New developments that can facilitate the dialysis process for patients is a primary goal in the search of new filtering materials as well as measuring techniques to monitor water redistribution in the organism in the dialysis process–both cost-reducing and non-invasive methods. The development of feedback systems having the task of automatic control of dialysis and ultrafiltration process to optimize the treatment variables (in particular, the rate of ultrafiltration) requires sensor measurements [242,244,245,246] and computer modeling of the results [247].

Synthetic membranes of varying structure and chemical composition are used in contemporary dialyzing devices [249,250,251,252,253,254,255,256,257,258,259,260,261,262]. A prominent example is the filtration of blood in the artificial kidney systems by GAMBRO [250], Fresenius [251], Asahi Kasei [252], Nephros [253], also see in [249,254]. Transport of water through innovative polymer membranes with the improved sieving characteristics is a fundamental aim of dialysis [248]. Simultaneously, the important research direction is to study possible allergic reactions of patients on dialyzing membranes material [254,260,261].

Artificial HD membranes are very different in morphology of both skin-layer and core-layer. Modern nanotechnologies permit the fabrication of membranes with given porosity properties and the performance manipulation in the process of membrane preparation [262]. To find the best structure-geometry for the optimization of pore functional shapes, a predictive modeling is needed [144,151,152,162,263]. Another help is the ‘flows-from-structure’’ computerized treatment of micro-tomography images and calculation of permeability value. Such analysis in combination with the experimental data on x-ray tomography has been done in [264] providing the example of evaluation of flow permeability of porous materials with higher precision where the error of the numerical calculations due to the finite resolution of the tomography images was found not essentially larger than the reported experimental values.

Biofouling [167,263] is another important factor in design of improved HD filters [166,168,265,266]. In the latter, proteins–filter surface interaction has a vital role. The most important example is a heparin coating [173,174,175]. Since the exposure of blood to the surface of the synthetic HD filter leads to the adsorption of proteins [266], the interaction of polymers and biomolecules are of key importance for biocompatibility and adhesion to the HD filters. Several aspects at macromolecular translocation across the artificial membrane pores should be taken into account: size and geometry of pores (for example, allow the passage of $\beta_{2}$ microglobulin [159]) and possible flexibility of the molecules during the channel passage.

Models of permeability and fouling of filters should provide an insight into the mechanism of pore blocking. In this view, the theories of porous materials which model the structure of the materials as random voids interconnected with necks in a 3D network [149] and include the coverage and blockage of pore entrance (i.e., fouling) in parallel with percolation phenomena could give us useful hints for the analysis. The notions of ’tortuosity’ and “porosity” [176] introduced in the theory as universal physical characteristics of the material independent on its chemical composition. This approach opens a way for a unified theoretical basis for the transport description in such a complex systems as biological and artificial membrane filters used in dialysis. Theoretical modeling of hindered diffusion is an extensive research area which cannot be covered in one short review. The example of theoretical models of hindered molecular transport and pore blocking could be found, for example, in [144,145,146,151].

The conventional HD treatment is an expensive procedure. What is the alternative method for patients with a kidney failure? This is a very reasonable question for developing countries due to the restrictive budget of clinics, limited medical facilities or inaccessibility of hemodialysis equipment.

A new idea of how to remove the toxins during the blood filtration has been suggested in Japan International Center for Materials Nanoarchitectonics [267]. The innovation for blood purification is an electrospun network of polymer nanofibers filled with zeolite particles. Due to the microporous structure of zeolites, the particles can efficiently adsorb uremic toxins from the blood.

Modeling adsorption in porous media can provide essential support to the experimental studies. In this direction, the calculations based on empirical equations were suggested in [268] where the effects of pore curvature were studied for the adsorption in microporous zeolites. Another help is the application of lattice density functional methods [269] extending the theory from micro–to mesoporous media.

Novel materials allow researchers to tune electromechanically the permeation of solutes across the artificial nanoporous membranes. Recently, graphene-embedded crown ether pores compatible with the graphene lattice have been reported elsewhere [270]. The new graphene-based nanoporous membranes show a variety of potential applications, in particular, in control of ionic flows and sieving by changing the channel (pore) configuration, from enhanced permeability to complete closing [270]. The artificial nanoporous membranes open a way to study also the role of electrostatics and mechanical deformations involved in sieving of flexible charged molecular probes.

## 9. Quantification of Dialysis: The Summary of Theoretical Concepts

Main goal of our work is to provide an integrated view on theoretical physics concepts behind a molecular transport across dialysis membranes. Since quantification of dialysis was and remains to be a prime target for the researchers, the review highlights the key importance of mathematical modeling, a vital analytical tool increasing the quality of dialysis control.

The review presents both hemodialysis and peritoneal dialysis modeling. The paper examines trends in mathematical modeling, which seek to solve the persistent problem of the adequacy of dialysis prescription. One of the most important notion, the “concept of clearance” has been presented by Sargent and Gotch [271,272,273,274] to the modeling of urea kinetics. The experimentally measured parameter (a measure of dialysis), K·t/V, is a ratio of dialyzer clearance (multiplied to the time of dialysis session) to the volume of urea distribution in the patient’s body. To estimate correctly this parameter, the essential efforts have been made by Flanigan, Fangman and Lim [275], Krediet and co-workers [88,89,92], Popovich and co-workers [276], Daugirdas and co-workers [277,278,279,280] and Waniewsky and co-workers [281,282,283,284,285,286,287,288,289,290]. An extensional theoretical analysis of PD (and HD) transport based on non-equilibrium thermodynamics has been developed in a number of theoretical works by Waniewsky [281,282] and co-workers [283,284,285,286,287,288,289,290]. In there [285,286,287,288,289,290], the kinetic modeling of dialysis originated from one pool–, two pool–and multiple pool theory of Popovich and co-workers [276], was generalized to calculate sieving coefficients and time–course for the fractional volume fluid fluxes and albumin in PD transport [290]. The exact mathematical solutions obtained in the so-called spatially-distributed model [290] have been supplemented with numerical simulations for the tissue in contact with peritoneum. New mathematical tools for controlled dialysis include also a web-based program and advanced kinetic modeling developed in [291,292,293].

Filtration in dialysis is a multi-parametric process guided with a complex kinetics of water and solutes penetrating through porous membranes of composite structure. It is a vast field and the research literature is voluminous. For this reason, in the present review we focused on selected models only. In particular, we considered the two pore model, three pore model (TPM) and the extended TPM in dialysis applications. Although the peritoneal wall has an extremely sophisticated labyrinthine structure, the TPM model was shown can successfully reflect main features of the peritoneal transport and quantitatively describe the physical mechanism of transport of water and solutes in agreement with experiments. In particular, it was demonstrated how the extended TMP model can be used in optimizing automated peritoneal dialysis [141].

The consideration of tortuous pathways structure was not included in above mentioned models. The useful notions of tortuosity and porosity have been introduced in the theory of hindered diffusion (see, for the review, in [176], also [144,145,146,147,148,149,150,151,152,153,154]). Specifically, it was shown that the molecular transport across tortuous pores has peculiarities for the cake layers formed at fouling of the dialysis filters [144,154]. The tortuosity and porosity are crucial physical characteristics of hindered diffusion through cake layers [144,149,153,154,162]. The examples of molecular models for tortuous membranes are briefly discussed in the second part of the present review.

The essential effort of current review is to bring together physical concepts of non-equilibrium thermodynamics and electrical conductivity–diffusion analogy and show how the selected mathematical models presented here emerge, follow and further develop these fundamental theoretical approaches in practical applications in dialysis. One should emphasize the importance of an analogy between the electrical conductivity and diffusion in porous media. This analogy has already called out by Lord Rayleigh in his work “On the influence of obstacles…” [183] and then, effectively used by Von Carman [178] and in a large number of research works on diffusion through porous materials (see, for the review, a very comprehensive book of Dullien [176]). In addition, there is a deep influence of the theory of dielectric “mixtures’’ originally developed in Maxwell [180], Lord Rayleigh [183], Wagner [184], Bruggeman [294] works and later developed by Hanai [295], Hanai & Kozumi [296], Looyega [297], De La Rue & Tobias [298], Fricke [299] and developments based on this theory on a wide range of disciplines (see, for example, [298,299,300,301,302,303,304]). In particular, this theory has provided basis for the theory of bioimpedance analysis (BIA) and bioimpedance spectroscopy (BIS) widely used in hemodialysis for the assessment of total body water, as well as intracellular and extracellular water [245,302,303,304,305,306]. The latter parameters are crucial for the correct dialysis performance and the so-called “dry weight” evaluation [304,305,306].

When discussing the most influential theoretical concepts in dialysis, the Starling [307] equation and the Staverman [308] reflection coefficient should be me mentioned first of all. Starling equation is one of the kinetic equations in the Kedem-Katchalsky theory of fluid transport across a semi-permeable membrane Introduced in a simple phenomenological form of a linear equation relating a fluid filtration flux and a conjugated force due to the difference in hydrostatic and oncotic pressures (often called a Sterling force), this equation provides a mathematical description of filtration across capillary wall. New experimental results [309] reported the observed discrepancy between the “ideal” Starling’s kinetics and the filtration across capillaries revealing more complex scenario and the important role of membrane sub-structures.

Another comment is the universality of both fundamental physical theories. The non-equilibrium thermodynamics framework and the conductivity-diffusion analogy demonstrate general physical principles of mass transport across porous membranes. The kinetic equations for conductivity (or resistivity) and diffusion are linear relations between the conjugated fluxes and generalized forces while the kinetic coefficients obey the Onsager’s principle of symmetry. Kedem-Katchalsky theory together with Onsager’s reciprocal relations also provide basic equations for the description of transport of water across model and biological cell membranes. (The osmotic balance is the crucial condition for the survival of blood cells in hemodialysis. While the analysis of osmotic behavior of blood cells and transport across their membranes is beyond the scope of current review, we provided a detailed Kedem–Katchalsky–based description of thermodynamics of water and solute transport through a porous capillary wall which could model a blood vessel, glomerular membrane or a synthetic polymer filter for dialysis.)

The last but not least research question is the adequate theoretical analysis of blood cell shapes at contact with biomedical materials, in particular, with the surface of polymer filters in blood purification. The curvature elasticity approach pioneered by Canham and Helfrich opened a way to the powerful theoretical developments which allow researchers to quantify shape changes caused by various factors, from osmotic perturbations to surface adhesion. The curvature elasticity approach based on Helfrich’s expression for the bending energy of the membrane is a generalization of bending energy of rods considered in the framework of theory of elasticity [310].

## Abbreviations

APD	automated peritoneal dialysis
AQP	aquaporin
CA	cellulose acetate
CKD	chronical kidney disease
DFR	dialysate flow rate
FWT	free water transport
GFB	glomerular filtration barrier
GBM	glomerular basement membrane
HD	hemodialysis
HDF	hemodiafiltration
MRI	magnetic resonance imaging
PA	polyamide
PD	peritoneal dialysis
PET	peritoneal equilibration test
PES	polyethersulfone
PSf	polysulfone
RBC	red blood cell
RRT	renal replacement therapy
TPM	three-pore model
UF	ultrafiltration

## References

[1] Alexander S.R., Cochat P. (2012). Notes on the history of dialysis therapy in children. Pediatric Dialysis.

[2] Paskalev D.N. (2001). Georg Haas (1886–1971): The Forgotten Hemodialysis Pioneer. Dial. Transplant..

[3] Schmaldienst S., Hörl W.H., Hörl W.H., Koch K.M., Lindsay R.M., Ronco C., Winchester J.F. (2004). The biology of hemodialysis. Replacement of Renal Function by Dialysis.

[4] Pregl F. (1914). Beitrage zur Methodik des Dialysierverfahrens von E.Abderhalden. Fermentforschung.

[5] Shinaberger J.H. (2001). Quantitation of dialysis: Historical perspective. Semin. Dial..

[6] Abel J.J., Rowntree L.G., Turned B.B. (1914). On the removal of diffusible substances from the circulation blood of living animals by dialysis. J. Pharmacol. Exp. Theor..

[7] Kolff W., Berk H., Welle M., van der Ley A.J.W., van Dijk E.C., van Noordwijk J. (1944). The artificial kidney: A dialyser with a great area. Acta Med. Scand..

[8] Haas G. (1952). Uber die kunstliche Niere. Dt Med. Wochenschr..

[9] Stegmayr B. (2016). Utvecklingen av hemodialysis i Sverige. Svensk Njurmedicinsk Förening 50 år. Berättelser om Dialys och Njursjukvård i Sverige.

[10] Hakim R.M., Breillatt J., Lazarus J.M., Port F.K. (1984). Complement activation and hypersensitivity reactions to dialysis membranes. N. Engl. J. Med..

[11] Hakim R.M., Fearn D.T., Lazarus J.M., Perzanowski C.S. (1984). Biocompatibility of dialysis membranes: Effects of chronic complement activation. Kidney Int..

[12] Henderson L.W., Cheung A.K., Chenoweth D.E. (1983). Choosing a Membrane. Am. J. Kid. Dis..

[13] Yamashita A.C., Sakurai K., Suzuki H. (2015). Dialysis membranes–physico-chemical structures and features. Updates in Hemodialysis.

[14] Babb A.L., Strand M.J., Uvelli D.A., Milutinovic J., Scribner B.H. (1975). Quantitative description of dialysis treatment: A dialysis index. Kidney Int. Suppl..

[15] Tenckhoff H., Curtis F.K. (1970). Experience with maintainance peritoneal dialysis in the home. Trans. Am. Soc Artif. Intern. Organs.

[16] Arkill K.P., Qvortrup K., Starborg T., Mantell J.M., Knupp C., Michel C.C., Harper S.J., Salmon A.H.J., Squire J.M., Bates D.O. (2014). Resolution of the three dimensional structure of components of the glomerular filtration barrier. BMC Nephrol..

[17] Salmon A.H.J., Neal C.R., Harper S.J. (2009). New aspects of glomerular filtration barrier structure and function: Five layers (at least) not three. Curr. Opin. Nephrol. Hypertens..

[18] Haraldsson B., Jeansson M. (2009). Glomerular filter barrier. Curr. Opin. Nephrol. Hypertens..

[19] Jarad G., Cunningham J., Shaw A.S., Miner J.H. (2006). Proteinuria precedes podocytes abnormalities in Lamb mice, implicating the glomerular basement membrane as an albumin barrier. J. Clin. Investig..

[20] Jarad G., Miner J.H. (2009). Update on the glomerular filtration barrier. Curr. Opin. Nephrol. Hypertens..

[21] Abrahamson D.R., Wang R., Vize P.D., Woolf A.S., Bard J.B.L. (2003). Development of the glomerular capillary and its basement membrane. The Kidney. From Normal Development to Congenital Disease.

[22] Leung J.C.K., La K.N., Tang S.C.W. (2014). Crosstalk between podocytes and tubular epithelial cells. Podocytopathy. Contrib. Nephrol..

[23] Holthöfer H. (2007). Molecular architecture of the glomerular slit diaphragm: Lessons learnt for a better understanding of disease pathogenesis. Nephrol. Dial. Transplant..

[24] Satchell S.C., Braet F. (2009). Glomerular endothelial cell fenestrations: An integral component of the glomerular filtration barrier. Am. J. Physiol. Ren. Physiol..

[25] Sekulic M., Sekulic S.P. (2013). A compendium of urinary biomarkers indicative of glomerular podocytopathy. Pathol. Res. Int..

[26] Hackl M.J., Burford J.L., Villanueva K., Lam L., Suszták K., Schermer B., Benzing T., Peti-Peterdi J. (2013). Tracking the fate of glomerular epithelial cells in vivo using serial multiphoton imaging in novel mouse models with fluorescent lineage tags. Nat. Med..

[27] Peti-Peterdi J., Kidokoro K., Riquier-Brison A. (2015). Novel in vivo techniques to visualize kidney anatomy and function. Kidney Int..

[28] Chagnac A., Herskovitz P., Weinstein T., Elyashiv S., Hirsh J., Hammel I., Gafter U. (1999). The peritoneal membrane in peritoneal dialysis patients: Estimation of its functional surface area by applying stereologic methods to computerized tomography scans. J. Am. Soc. Nephrol..

[29] Heilmann M., Neudecker S., Wolf I., Gubhaju L., Sticht C., Schock-Kusch D., Kriz W., Bertram J.F., Schad L.R., Gretz N. (2011). Quantification of glomerular number and size distribution in normal rat kidneys using magnetic resonance imaging. Nephrol. Dial. Transplant..

[30] Layton A.T. (2013). Mathematical modeling of kidney transport. WIREs Syst Biol Med..

[31] Layton A.T. (2012). Modeling transport and flow regulatory mechanisms of the kidney. Int. Sch. Res. Netw. ISRN Biomath..

[32] Deen W.M. (1987). Hindered transport of large molecules in liquid-filled pores. AIChE J..

[33] Rippe B., Haraldsson B. (1994). Transport of macromolecules across microvascular walls: The two-pore theory. Physiol. Rev..

[34] Deen W.M. (2004). What determines glomerular capillary permeability?. J. Clin. Investig..

[35] Deen W.M., Bohrer M.P., Epstein N.B. (1981). Effects of molecular size and configuration on diffusion in microporous membranes. AIChE.

[36] Deen W.M., Bridges C.R., Brenner B.M., Myers B.D. (1985). Heteroporous model of glomerular size selectivity: Application to normal and nephrotic humans. Am. J. Physiol..

[37] Deen W.M., Lazzara M.J., Myers B.D. (2001). Structural determinants of glomerular permeability. Am. J. Physiol. Ren. Physiol..

[38] Edwards A. (2010). Modeling transport in the kidney: Investigating function and dysfunction. Am. J. Physiol. Ren. Physiol..

[39] Edwards A., Daniels B.S., Deen W.M. (1999). Ultrastructural model for size selectivity in glomerular filtration. Am. J. Physiol. Ren. Physiol..

[40] Comper W.D., Haraldsson B., Deen W.M. (2008). Resolved: Normal glomeruli filter nephrotic levels of albumin. J. Am. Soc. Nephrol..

[41] Haraldsson B., Nystrom J., Deen W.M. (2008). Properties of the glomerular barrier and mechanisms of proteinuria. Physiol. Rev..

[42] Russo L.M., Sandoval R.M., McKee M., Osicka T.M., Collins A.B., Brown D., Molitoris B.A., Comper W.D. (2007). The normal kidney filters nephrotic levels of albumin retrieved by proximal tubule cells: Retrieval is disrupted in nephrotic states. Kidney Int..

[43] Chen S., Wassenhove-McCarthy D.J., Yamaguchi Y., Holzman L.B., van Kuppevelt T.H., Jenniskens G.J., Wijnhoven T.J., Woods A.C., McCarthy K.J. (2008). Loss of heparan sulfate glycosaminoglycan assembly in podocytes does not lead to proteinuria. Kidney Int..

[44] Jeansson M., Haraldsson B. (2006). Morphological and functional evidence for an important role of the endothelial cell glycocalyx in the glomerular barrier. Am. J. Physiol. Ren. Physiol..

[45] Harvey S.J., Miner J.H. (2008). Revisiting the glomerular charge barrier in the molecular era. Curr. Opin. Nephrol. Hypertens..

[46] Greive K.A., Nikolic-Paterson D.J., Guimaraes M.A., Nikolovski J., Pratt L.M., Mu W., Atkins R.C., Comper W.D. (2001). Glomerular perselectivity factors are not responsible for the increase in fractional clearance of albumin in rat glomerulonephritis. Am. J. Pathol..

[47] Asgeirsson D., Venturoli D., Fries E., Rippe B., Rippe C. (2007). Glomerular sieving of three neutral polysaccharides, polyethylene oxide and bikunin in rat. Effects of molecular size and conformation. Acta Physiol..

[48] Asgeirsson D., Axelsson J., Rippe C., Rippe B. (2009). Similarity of permeabilities for Ficoll pullulan, charge-modified albumin and native albumin across the rat peritoneal membrane. Acta Physiol..

[49] Slattery C., Lee A., Zhang Y., Kelly D.J., Thorn P., Nikolic-Paterson D.J., Tesch G.H., Poronnik P. (2008). In vivo visualization of albumin degradation in the proximal tubule. Kidney Int..

[50] Axelsson J., Sverrisson K., Rippe A., Fissell W., Rippe B. (2012). Reduced diffusion of charge-modified, conformationally intact anionic Ficoll relative to neutral Ficoll across the rat glomerular filtration barrier. J. Membr. Sci..

[51] Axelsson J., Öberg C.M., Rippe A., Krause B., Rippe B. (2012). Size-selectivity of a synthetic high-flux and a high cut-off dialyzing membrane compared to that of the rat glomerular filtration barrier. J. Membr. Sci..

[52] Venturoli D., Rippe B. (2005). Ficoll and dextran vs. globular proteins as probes for testing glomerular permselectivity: Effects of molecular size, shape, charge, and deformability. Am. J. Physiol. Ren. Physiol..

[53] Rippe C., Asgeirsson D., Venturoli D., Rippe A., Rippe B. (2006). Effects of glomerular filtration rate on Ficoll sieving coefficients (θ) in rats. Kidney Int..

[54] Öberg C.M., Rippe B. (2013). Quantification of the electrostatic properties of the glomerular filtration barrier modeled as a charged fiber matrix separating anionic from neutral Ficoll. Am. J. Ren. Physiol..

[55] Öberg C.M., Rippe B. (2014). A distributed two-pore model: Theoretical implications and practical application to the glomerular sieving of Ficoll. Am. J. Physiol. Ren. Physiol..

[56] Rippe B., Öberg C.M. (2015). Counterpoint: Defending pore theory. Perit. Dial. Int..

[57] Rippe B., Davies S. (2011). Permeability of peritoneal and glomerular capillaries: What are the differences according to pore theory?. Perit. Dial. Int..

[58] Rippe B. (1993). A three-pore model of peritoneal transport. Perit. Dial. Int..

[59] Devuyst O., Rippe B. (2014). Water transport across the peritoneal membrane. Kidney Int..

[60] Devuyst O., Ni J. (2005). Aquaporin-1 in the peritoneal membrane: Implications for water transport across capillaries and peritoneal dialysis. Biochim. Biophys. Acta.

[61] Zhang W., Freichel M., van der Hoeven F., Nawroth P.P., Katus H., Kälble F., Zitron E., Schwenger V. (2016). Novel endothelial cell-specific AQP1 knockout mice confirm the crucial role of endothelial AQP1 in ultrafiltration during peritoneal dialysis. PLoS ONE.

[62] Agre P., Kozono D. (2003). Aquaporin water channels: Molecular mechanisms for human diseases. FEBS Lett..

[63] Endeward V., Musa-Aziz R., Cooper G.J., Chen L.M., Pelletier M.F., Virkki L.V., Supuran C.T., King L.S., Boron W.F., Gros G. (2006). Evidence that aquaporin1 is a major pathway for CO_2_ transport across the human erythrocyte membrane. FASEB J..

[64] Ripoche P., Goossens D., Devuyst O., Gane P., Colin Y., Verkman A.S., Cartron J.P. (2006). Role of RhAG and AQP1 in NH3 and CO_2_ gas transport in red cell ghosts: A stopped-flow analysis. Transf. Clin. Biol..

[65] Missner A., Kügler P., Saparov S.M., Sommer K., Mathai J.C., Zeidel M.L., Pohl P. (2008). Carbon dioxide transport through membranes. J. Biol. Chem..

[66] Hub J.S., Grubmüller H., de Groot B.L. (2009). Dynamics and energetics of permeation through aquaporins. What do we learn from molecular dynamics simulations?. Handb. Exp. Pharmacol..

[67] Verkman A.C. (2011). Aquaporins at a glance. J. Cell. Sci..

[68] Verkman A.C. (2008). Dissecting the role of aquaporins in renal pathophysiology using transgenic mice. Semin. Nephrol..

[69] Noda Y., Sohara E., Ohta E., Sasaki S. (2010). Aquaporins in kidney pathophysiology. Nat. Rev. Nephrol..

[70] Day R.E., Kitchen P., Owen D.S., Bland C., Marshall L., Conner A.C., Bill R.M., Conner M.T. (2014). Human aquaporins: Regulators of transcellular water flow. Biochim. Biophys. Acta.

[71] Agre P. (2004). Aquaporin water channels (Nobel Lecture). Angew. Chem. Int. Ed. Engl..

[72] Verkman A.C. (2006). Roles of aquaporins in kidney revealed by transgenic mice. Semin. Nephrol..

[73] Nielsen S., Frøkiær J., Marples D., Kwon T.H., Agre P., Kneppe M.A. (2002). Aquaporins in the kidney: From molecules to medicine. Physiol. Rev..

[74] Ni J., Verbavatz J.-M., Rippe A., Boisdé I., Moulin P., Rippe B., Verkman A.S., Devuyst O. (2006). Aquaporin-1 plays an essential role in water permeability and ultrafiltration during peritoneal dialysis. Kidney Int..

[75] Devuyst O., Yool A.J. (2010). Aquaporin-1: New developments and perspectives for peritoneal dialysis. Perit. Dial..

[76] Denker B.M., Smith B.L., Kuhajda F.P., Agre P. (1988). Identification, purification, and partial characterization of a novel Mr 28,000 integral membrane protein from erythrocytes and renal tubules. J. Biol. Chem..

[77] Brown D. (2017). The discovery of water channels (aquaporins). Ann. Nutr. Metab..

[78] Morelle J., Devuyst O. (2015). Water and solute transport across the peritoneal membrane. Curr. Opin. Nephrol. Hypertens..

[79] Morelle J., Amadou Sow A., Vertommen D., Jamar F., Rippe B., Devuyst O. (2014). Quantification of osmotic water transport in vivo using fluorescent albumin. Am. J. Physiol. Ren. Physiol..

[80] Yang B., Folkesson H.G., Yang J., Matthay M.A., Verkman A.S. (1999). Reduced osmotic water permeability of the peritoneal barrier in aquaporin-1 knockout mice. Am. J. Physiol..

[81] Flessner M. (2006). Water-only pores and peritoneal dialysis. Kidney Int..

[82] King L.S., Moon C., Agre P. (1996). Aquaporin-1 water channel protein in lung: Ontogeny, steroid-induced expression, and distribution in rat. J. Clin. Investig..

[83] Stoenoiu M.S., Ni J., Verkaeren C., Debaix H., Janas J.C., Lameire N., Varbavatz J.-M., Devuyst O. (2003). Corticosteroids induce expression of aquaporin-1 and induce transcellular water transport in rat peritoneum. J. Am. Soc. Nephrol..

[84] Zomot E., Bakan A., Shrivastava I.H., DeChancie J., Lezon T.R., Bahar I., Roux B. (2011). Sodium-coupled Secondary Transporters: Insights from Structure-based Computations. Molecular Machines.

[85] Umenishi F., Schrier R.W. (2003). Hypertonicity-induced aquaporin-1 (AQP1) expression is mediated by the activation of MAPK pathways and hypertonicity-responsive element in the AQP1 gene. J. Biol. Chem..

[86] Belkacemi L., Beall M.H., Magee T.R., Pourtemou M., Ross M.G. (2008). AQP1 gene expression is upregulated by arginine vasopressin and cyclic AMP agonists in trophoblast cells. Life Sci..

[87] Bouley R., Palomino Z., Tang S.S., Nunes P., Kobori H., Lu H.A., Shum W.W., Sabolic I., Brown D., Ingelfinger J.R. (2009). Angiotensin II and hypertonicity and hypertonicity modulate proximal tubular aquaporin 1 expression. Am. J. Physiol. Ren. Physiol..

[88] Krediet R.T., Gokal R., Khanna R., Krediet R.T., Nolph K.D. (2000). The physiology of peritoneal solute transport and ultrafiltration. Textbook of Peritoneal Dialysis.

[89] Cnossen T.T., Smit W., Konings C.J.A.M., Kooman J.P., Leunissen K.M., Krediet R.T. (2009). Quantification of free water transport during the peritoneal equilibration test. Perit Dial. Int..

[90] Twardowski Z.J., Nolph K.D., Khanna R., Prowant B.F., Ryan L.P., Moore H.L., Nielsen M.P. (1987). Peritoneal equilibration test. Perit. Dial. Bull..

[91] Twardowski Z.I. (1989). Clinical value of standardized equilibration tests in CAPD patients. Blood Purif..

[92] Krediet R.T., Lindholm B., Rippe B. (2000). Pathophysiology of peritoneal membrane failure. Perit. Dial. Int..

[93] LaMilia V., Di Filippo S., Crepaldi M., Del Vecchio L., Dell’oro C., Andrulli S., Locatelli F. (2005). Mini-peritoneal equilibration test: A simple and fast method to assess free water and small solute transport across the peritoneal membrane. Kidney Int..

[94] Parikova A., Watske S., Struijk D.G., Machteld M., Krediet R.T. (2005). The contribution of free water transport and small pore transport to the total fluid removal in peritoneal dialysis. Kidney Int..

[95] Rippe B., Stelin G. (1989). Simulations of peritoneal solute transport during CAPD. Applications of two-pore formalism. Kidney Int..

[96] Asghar R.B., Davies S.J. (2008). Pathways of fluid transport and reabsorption across the peritoneal membrane. Kidney Int..

[97] Rippe B., Venturoli D. (2008). Fluid loss from the peritoneal cavity by back-filtration through the small pores of the three-pore model. Kidney Int..

[98] Nielsen S., DiGiovanni S.R., Christensen E.I., Knepper M.A., Harris H.W. (1993). Cellular and subcellular immunolocalization of vasopressin regulated water channel in rat kidney. Proc. Natl. Acad. Sci. USA.

[99] Nielsen S., Kwon T.H., Christensen B.M., Promeneur D., Frøkiær J., Marples D. (1999). Physiology and pathophysiology of renal aquaporins. J. Am. Nephrol..

[100] Kishore B.K., Mandon B., Oza N.B., DiGiovanni S.R., Coleman R.A., Ostrowski N.L., Wade J.B., Knepper M.A. (1996). Rat renal arcade segment expresses vasopressin-regulated water channel and vasopressin V2 receptor. J. Clin. Investig..

[101] Me P., Taylor A. (1989). Effect of nocodazole on the water permeability response to vasopressin in rabbit collecting tubules perfused in vitro. J. Physiol. (Lond.).

[102] Knepper M.A. (1997). Molecular physiology of urinary concentrating mechanism: Regulation of aquaporin water channels by vasopressin. Am. J. Physiol. Ren. Physiol..

[103] Loffing J., Loffing-Cueni D., Macher A., Hebert S.C., Olson B., Knepper M.A., Rossier B.C., Kaissling B. (2000). Localization of epitheliasodium channel and aquaporin-2 in rabbit kidney cortex. Am. J. Physiol. Ren. Physiol..

[104] Aguirre A.R., Abensu H. (2014). Physiology of fluid and solute transport across the peritoneal membrane. J. Bras. Nefrol..

[105] Katz M.A., Schaeffer R.C., Gratrix M., Mucha D., Cárbajal J. (1999). The glomerular barrier fits a two –pore-and-fiber-matrix model: Derivation and physiologic test. Microvasc. Res..

[106] Rippe B. (2008). Free water transport, small pore transport and the osmotic pressure gradient three-pore model of peritoneal transport. Nephrol. Dial. Transplant..

[107] Fu B.M., Curry F.E., Weinbaum S. (1995). A diffusion wake model for tracer ultrastructure-permeability studies in microvessels. Am. J. Physiol..

[108] Henry C.B.S., Duling B.R. (2000). TNF-alpha increases entry of macromolecules into luminal endothelial cell glycocalyx. Am. J. Physiol..

[109] Kedem O., Katchalsky A. (1958). Thermodynamic analysis of the permeability of biological membranes to nonelectrolytes. Biochim. Biophys. Acta.

[110] Kedem O., Katchalsky A. (1961). A physical interpretation of the phenomenological coefficients of membrane permeability. J. Gen. Physiol..

[111] Kedem O., Katchalsky A. (1963). Permeability of composite membranes. Trans. Faraday Soc..

[112] Onsager L. (1931). Reciprocal relations in irreversible processes. Part, I. Phys. Rev..

[113] Kotyk A., Janáček K. (1975). Cell membrane Transport. Principles and Techniques.

[114] Landau L.D., Lifshitz E.M. (2009). Statistical physics. Course of Theoretical Physics.

[115] Garlick D.G., Renkin E.M. (1970). Transport of large molecules from plasma to interstitial fluid and lymph in dogs. Am. J. Physiol. Scand..

[116] Renkin E.M. (1964). Transport of large molecules across capillary walls. Physiologist.

[117] Renkin E.M. (1985). Capillary transport of macromolecules: Pores and other endothelial pathways. J. Appl. Physiol..

[118] Tencer J., Frick I.M., Öqvist B.W., Alm P., Rippe B. (1998). Size-selectivity of the glomerular barrier to high molecular weight proteins: Upper size limitations of shunt pathways. Kidney Int..

[119] Bark B.P., Öberg C.M., Grände P.O. (2013). Plasma volume expansion by 0.9% NaCl during sepsis/SIRS, after hemorrhage, and during a normal state. Shock.

[120] Miller N.E., Michel C.C., Nanjee M.N., Olszewski W.L., Miller I.P., Hazell M., Olivecrona G., Sutton P., Humphreys S.M., Frayn K.N. (2011). Secretion of adipokines by human adipose tissue in vivo: Partitioning between capillary and lymphatic transport. Am. J. Physiol. Endocrinol. Metab..

[121] Taylor A.E., Granger D.N., Brace R.A. (1977). Analysis of lymphatic protein flux data. I. Estimation of the reflection coefficient and permeability surface area product for total protein. Microvasc. Res..

[122] Ibrahim R., Nitsche J.M., Kasting G.B. (2012). Dermal clearance model for epidermal bioavailability calculations. J. Pharm. Sci..

[123] Arturson G., Groth T., Grotte G. (1971). Human glomerular membrane porosity and filtration pressure: Dextrane clearance data analysed by theoretical models. Clin. Sci..

[124] Blouch K., Deen W.M., Fauvel J.P., Bialek J., Derby G., Myers B.D. (1985). Molecular configuration and glomerular size selectivity in healthy and nephrotic humans. Am. J. Physiol. Ren. Physiol..

[125] Rippe B., Haraldsson B. (1987). Fluid and protein fluxes across small and large pores in the microvasculature. Application of two-pore equations. Acta Physiol. Scand..

[126] Renkin E.M. (1979). Relation of capillary morphology to transport of fluid and large molecules: A review. Acta Physiol. Scand. Suppl..

[127] Renkin E.M. (1986). Some consequences of capillary permeability to macromolecules: Starling’s hypothesis reconsidered. Am. J. Physiol..

[128] Renkin E.M., Joyner W.L., Sloop C.H., Watson P.D. (1977). Influence of venous pressure on plasma-lymph transport in the dog’s paw: Convective and dissipative mechanisms. Microvasc. Res..

[129] Renkin E.M., Watson P.D., Sloop W.M., Curry F.E. (1977). Transport pathways for fluid and large molecules in miscovascular endothelium of the dog’s paw. Microvasc. Res..

[130] Perl W. (1975). Convection and permeation of albumin between plasma and interstitium. Microvasc. Res..

[131] Lund U., Rippe A., Venturoli D., Tenstad O., Grubb A., Rippe B. (2003). Glomerular filtration rate dependence of sieving of albumin and some neutral proteins in rat kidneys. Am. J. Physiol. Ren. Physiol..

[132] Öberg C.M., Groszek J.J., Roy S., Fissell W.H., Rippe B. (2017). A distributed solute model: An extended two-pore model with application to the glomerular sieving of Ficoll. Am. J. Physiol. Ren. Physiol..

[133] Zydney A.L., Aimar P., Meireles M., Pimbley J.M., Belfort G. (1994). Use of the log-normal probability density function to analyze membrane pore size distributions: Functional forms and discrepancies. J. Membr. Sci..

[134] Haraldsson B. (1995). Assessing the peritoneal dialysis capacities of individual patients. Kidney Int..

[135] Rippe B., Stelin G., Haraldsson B. (1991). Computer simulations of peritoneal fluid transport in CAPD. Kidney Int..

[136] Devuyst O., Goffin E. (2008). Water and solute transport in peritoneal dialysis: Models and clinical applications. Nephrol. Dial. Transplant..

[137] Vonesh E.F., Rippe B. (1992). Net fluid absorption under membrane transport models of peritoneal dialysis. Blood Purif..

[138] Rippe B., Venturoli D., Simonsen O., de Arteaga J. (2004). Fluid and electrolyte transport across the peritoneal membrane during CAPD according to the three-pore model. Perit. Dial. Int..

[139] Freida P., Galach M., Divino Filho J.C., Werynski A., Lindholm B. (2007). Combination of crystalloid (glucose) and colloid (icodextrin) osmotic agents markedly enhances peritoneal fluid and solute transport during the long PD dwell. Perit. Dial. Int..

[140] Rippe B., Levin L. (2000). Computer simulations of ultrafiltration profiles for an icodextrin-based peritoneal fluid in CAPD. Kidney Int..

[141] Öberg C.M., Rippe B. (2017). Optimizing automated peritoneal dialysis using an extended 3-pore model. Kidney Int. Rep..

[142] Rippe B., Stelin G., Ahlmen J., Maher J.F., Winchester J.F. (1986). Lymph flow from the peritoneal cavity in CAPD patients. Frontiers in Peritoneal Dialysis.

[143] Rippe B., Krediet R.T. (1994). Peritoneal physiology-transport of solutes. The Textbook of Peritoneal Dialysis.

[144] Polyakov Y.S., Zydney A.L. (2013). Ultrafiltration membrane performance: Effects of pore blockage/constriction. J. Membr. Sci..

[145] Hermia J. (1982). Constant pressure blocking filtration laws–application to power-law non-Newtonian fluids. Trans. Inst. Chem. Eng.–Lond..

[146] Hermia J., Rushton A. (1985). Blocking filtration. Application to Non-Newtonian fluids. Mathematical Models and Design Methods in Solid-Liquid Separation.

[147] Santos A., Bedrikovetsky P. (2006). A stochastic model for particulate suspension flow in porous media. Transp. Porous Media.

[148] Santos A., Bedrikovetsky P., Fontoura S. (2008). Analytical micro model for size exclusion: Pore blocking and permeability reduction. J. Membr. Sci..

[149] Mehta A., Zydney A.L. (2005). Permeability and selectivity analysis for ultrafiltration membranes. J. Membr. Sci..

[150] Mehta A., Zydney A.L. (2006). Effect of membrane charge on flow and protein transport during ultrafiltration. Biotechnol. Progr..

[151] Zydney A.L., Oyama S.T., Stagg-Williams S.M. (2011). High performance ultrafiltration membranes: Pore geometry and charge effects, membrane science and technology. Inorganic, Polymeric, and Composite Membranes: Structure, Function, and Other Correlations, Part of Membrane Science and Technology Series.

[152] Kanani D.M., Fissel W.H., Roy S., Dubnisheva A., Fleishman A., Zydney A. (2010). Permeability–selectivity analysis for ultrafiltration: Effect of pore geometry. J. Membr. Sci..

[153] Armatas G.S., Salmas C.E., Louloudi M., Androutsopoulos G.P., Pomonis P.J. (2003). Relationships among pore size, connectivity, dimensionality of capillary condensation, and pore structure tortuosity of functionalized mesoporous silica. Langmuir.

[154] Armatas G.S. (2006). Determination of the effects of the pore size distribution and pore connectivity distribution on the pore tortuosity and diffusive transport in model porous networks. Chem. Eng. Sci..

[155] Ronco C., Ghezzi P.M., Bowry S.R., Hörl W.H., Koch K.M., Lindsay R.M., Ronco C., Winchester J.F. (2004). Membranes for hemodialysis. Replacement of Renal Function by Dialysis.

[156] Aoyagi S., Abe K., Yamagishi T., Iwai H., Yamaguchi S., Sunohara T. (2017). Evaluation of blood adsorption onto dialysis membranes by time-of-flight secondary ion mass spectrometry and near-filed infrared microscopy. Anal. Bioanal. Chem..

[157] Ochoa N.A., Prádanos P., Palacio L., Pagliero C., Marchese J., Hernández A. (2001). Pore size distributions based on AFM imaging and retention of multidisperse polymer solutes: Characterisation of polyethersulfone UF membranes with dopes containing different PVP. J. Membr. Sci..

[158] Feinberg B.J., Hsiao J.C., Park J., Zydney A.L., Fissell W.H., Roy S. (2018). Slit pores preferred over cylindrical pores for high selectivity in biomolecular filtration. J. Colloid Interface Sci..

[159] Ileri N., Létant S.E., Palazoglu A., Stroeve P., Tringe J.W., Faller R. (2012). Mesoscale simulations of biomolecular transport through nanofilters with tapered and cylindrical geometries. Phys. Chem. Chem. Phys..

[160] Hedayat A., Szpunar J., Kiran Kumar N.A.P., Peace R., Elmoselhi H., Shoker A. (2012). Morphological characterization of the polyflux 210H hemodialysis filter pores. Int. J. Nephrol..

[161] Ronco C., Clark W.R. (2018). Haemodialysis membranes. Nat. Rev. Nephrol..

[162] Kim A.S., Chen H. (2006). Diffusive tortuosity factor of solid and soft cake layers: A random walk simulation approach. J. Membr. Sci..

[163] Kokubo K.-I., Sakai K. (1998). Evaluation of dialysis membranes using a tortuous pore model. AlChE J..

[164] Kazuhiko I., Fukumoto K., Iwasaki Y. (1999). Modification of polysulfone with phospholipid polymer for improvement of the blood compatibility. Part 1. Surface characterization. Biomaterials.

[165] Xie B., Zhang R., Zhang H., Xu A., Deng Y., Ly Y., Deng F., Wei S. (2016). Decoration of heparin and bovine serum albumin on polysulfone membrane assisted via polydopamine strategy for hemodialysis. J. Biomater. Sci. Polym. Ed..

[166] Yua H., Caoa Y., Kanga G., Liua J., Lia M., Yuana Q. (2009). Enhancing antifouling property of polysulfone ultrafiltration membrane by grafting zwitterionic copolymer via UV-initiated polymerization. J. Membr. Sci..

[167] Wisniewski N., Moussy F., Reichert W.M. (2000). Characterization of implantable biosensor membrane biofouling. Fresenius J. Anal. Chem..

[168] Zhao W., He C., Huiyuan W., Baihai S., Shudong S., Changsheng Z. (2011). Improved antifouling property of polyethersulfone hollow fiber membranes using additive of poly(ethylene glycol) methyl ether-b-poly(styrene) copolymers. Ind. Eng. Chem. Res..

[169] Zhao W., Huang J., Fang B., Nie S., Yi N., Su B., Li H., Zhao C. (2011). Modification of polyethersulfone membrane by blending semi-interpenetrating network polymeric nanoparticles. J. Membr. Sci..

[170] Yin Z., Cheng C., Qin H., Nie C., He C., Zhao C. (2015). Hemocompatible polyethersulfone/polyurethane composite membrane for high-performance antifouling and antithrombotic dialyzer. J. Biomed. Mater. Res. Part B.

[171] Zhu L., Song H., Wang J., Xue L. (2017). Polysulfone hemodiafiltration membranes with enhanced antofouling and hemocompatibility modified by poly(vinyl pyrrolidone) via in situ cross-linked polymerization. Mater. Sci. Eng. C Mater. Biol. Appl..

[172] Rana D., Matsura T. (2010). Surface modifications for antifouling membranes. Chem. Rev..

[173] Hinrichs W.L.J., Ten Hoopen H.W.M., Engbers G.H.M., Feijen J. (1997). In vitro evaluation of heparinized Cuprophan hemodialysis membranes. J. Biomed. Mater. Res..

[174] Shen J.I., Winkelmayer W.C. (2012). Use and safety of unfractionated heparin for anticoagulation during maintenance hemodialysis. Am. J. Kidney Dis..

[175] Shen J.I., Montez-Rath M.E., Mitani A.A., Erickson K.F., Winkelmayer W.C. (2014). Correlates and variance decomposition analysis of heparin dosing for maintenance hemodialysis in older US patients. Pharmacoepidemiol. Drug Saf..

[176] Dullien F.A.L. (1992). Porous Media: Fluid Transport and Pore Structure.

[177] Kozeny J. (1927). Ueber kapillare Leitung des Wassers im Boden. Sitzungsberichte Wiener Akademie.

[178] Carman P.C. (1937). Fluid flow through granular beds. Trans. Inst. Chem. Eng. Lond..

[179] Klinkenberg L.J. (1951). Analogy between diffusion and electrical conductivity in porous rocks. Bull. Geol. Soc. Am..

[180] Maxwell J.C. (1881). A Treatise on Electricity and Magnetism.

[181] Landau L.D., Lishitz E.M. (1960). Electrodynamics of Continuous Media, Course of Theoretical Physics.

[182] Neale G.H., Nader W.K. (1973). Prediction of transport processes within porous media: Diffusive flow processes within a homogeneous swarm of spherical particles. AIChe J..

[183] Rayleigh L. (1892). On the influence of obstacles arranged in rectangular order upon the properties of a medium. Lond. Edinb. Dublin Philos. Mag. J. Sci..

[184] Wagner K.W. (1914). Erklärung der dielektrischen Nachwirkungsvorgänge auf Grund Maxwellscher Vorstellungen. Arch. Electrotechn..

[185] Hizi U., Bergman D.J. (2000). Molecular diffusion in periodic porous media. J. Appl. Phys..

[186] Mitra P.P., Sen P.N., Schwartz L.M. (1993). Short-time behavior of the diffusion coefficient as a geometrical probe of porous media. Phys. Rev. B.

[187] Bessis M. (1973). Living Blood Cells and Their Ultrastructure.

[188] Lipowsky R., Sackmann E. (1995). Structure and Dynamics of Membranes: From Cells to Vesicles.

[189] Evans E., Skalak R. (1980). Mechanics and Thermodynamics of Biomembranes.

[190] Discher D.E., Mohandas N., Evans E.A. (1994). Molecular maps of red-cell deformation—hidden elasticity and in-situ connectivity. Science.

[191] Grzhibovskis R., Krämer E., Bernhardt I., Kemper B., Zanden C., Repin N.V., Tkachuk B.V., Voinova M.V. (2017). Shape of red blood cells in contact with artificial surfaces. Eur. Biophys J..

[192] Zandén C., Voinova M., Gold J., Mörsdorf D., Bernhardt I., Liu J. (2012). Surface characterization of oxygen plasma treated electrospun polyurethane fibres and their interaction with red blood cells. Eur. Polymer J..

[193] Bernhardt I., Ivanova L., Langehanenberg P., Kemper B., von Bally G. (2008). Application of digital holographic microscopy to investigate the sedimentation of intact red blood cells and their interaction with artificial surfaces. Bioelectrochemistry.

[194] Canham P.B. (1970). The minimum energy of bending as a possible explanation of the biconcave shape of the human red blood cell. J. Theor. Biol..

[195] Deuling H.J., Helfrich W. (1976). The curvature elasticity of fluid membranes: A catalogue of vesicle shapes. J. Phys. France.

[196] Deuling H.J., Helfrich W. (1977). The theoretical explanation of the myelin shapes of red blood cells. Blood Cells.

[197] Deuling H.J., Helfrich W. (1976). Red blood cell shapes as explained on the basis of curvature elasticity. Biophys. J..

[198] Helfrich W. (1973). Elastic properties of lipid bilayers: Theory and possible experiments. Z. Naturforsch..

[199] Sheetz M.P., Singer S.J. (1974). Biological membranes as bilayer couples: A molecular mechanism of drug-erythrocyte interactions. Proc. Natl. Acad. Sci. USA.

[200] Svetina S., Brumen M., Žeks B. (1985). Lipid bilayer elasticity and the bilayer couple interpretation of red cell shape transformations and lysis. Stud. Biophys..

[201] Svetina S., Ziherl P. (2008). Morphology of small aggregates of red blood cells. Bioelectrochemistry.

[202] Larkin T.J., Kuchel P.W. (2010). Mathematical models of naturally “morphed” human erythrocytes: Stomatocytes and echinocytes. Bull. Math. Biol..

[203] Diez-Silva M., Dao M., Han J., Lim C.T., Suresh S. (2010). Shape and biomechanical characteristics of human red blood cells in health and disease. MRS Bull..

[204] Rudenko S.V. (2010). Erythrocyte morphological states, phases, transitions and trajectories. Biochim. Biophys. Acta.

[205] Seifert U. (1991). Adhesion of vesicles in two dimensions. Phys. Rev. A.

[206] Seifert U. (1997). Configurations of fluid membranes and vesicles. Adv. Phys..

[207] Muñoz S., Sebastián J.L., Sancho M., Álvarez G. (2014). Elastic energy of the discocyte–stomatocyte transformation. Biochim. Biophys. Acta.

[208] Lipowsky R., Seifert U. (1991). Adhesion of vesicles and membranes. Mol. Cryst. Liq. Cryst..

[209] Seifert U., Lipowsky R. (1990). Adhesion of vesicles. Phys. Rev. A.

[210] Hägerstrand H., Mrowczynska L., Salzer U., Prohaska R., Michelsen K.A., Kralj-Iglič V., Iglič A. (2006). Curvature-dependent lateral distribution of raft markers in the human erythrocyte membrane. Mol. Membr. Biol..

[211] Sackmann E., Bruinsma R.F. (2002). Cell adhesion as wetting transition?. ChemPhysChem..

[212] Sackmann E., Smith A.-S. (2014). Physics of cell adhesion: Some lessons from cell-mimetic systems. Soft Matter.

[213] Kozlov M.M., Markin V.S. (1984). A theory of osmotic lysis of lipid vesicles. J. Theor. Biol..

[214] Voinova M.V. (1998). Phase transitions in mesoscopic spherical membranes. Statistical Mechanics of Biocomplexity.

[215] Chabanon M., Ho J.C.S., Liedberg B., Parikh A.N., Rangamani P. (2017). Pulsatile lipid vesicles under osmotic stress. Biophys. J..

[216] Shibly A.S.U., Ghatak C., Karal S.M.A., Moniruzzaman M., Yamazaki M. (2016). Experimental estimation of membrane tension induced by osmotic pressure. Biophys J..

[217] Diz-Munõz A., Fletcher D.A., Weiner O.D. (2013). Use the force: Membrane tension as an organizer of cell shape and motility. Trends Cell. Biol..

[218] Sackmann E., Merkel R. (2010). Lehrbuch der Biophysik.

[219] Lipowsky R. (1998). From membranes to membrane machines. Statistical Mechanics of Biocomplexity.

[220] Sukumaran S., Seifert U. (2001). Influence of shear flow on vesicles near a wall: A numerical study. Phys. Rev. E.

[221] Waugh R.E. (1996). Elastic energy of curvature-driven bump formation on red blood cell membrane. Biophys. J..

[222] Iglič A., Kralj-Iglič V., Hägerstrand H. (1998). Stability of speculated red blood cells induced by intercalation of amphiphiles in cell membrane. Med. Biol. Eng. Comput..

[223] Mukhopadhyay R., Lim G.H.W., Wortis M. (2002). Echinocyte shape: Bending, stretching, and shear determine spicule shape and spacing. Biophys. J..

[224] Lim G.H.W., Wortis M., Mukhopadhyay R. (2002). Stomatocyte discocyte-echinocyte sequence of the human red blood cell: Evidence for the bilayer-couple hypothesis from membrane mechanics. Proc. Natl. Acad. Sci. USA.

[225] Kozlov M.M., Markin V.S., Chernyi G.C., Regirer S.A. (1990). Model of red blood cell membrane skeleton. Contemporary Problems of Biomechanics.

[226] Park Y.K., Best C.A., Badizadegan K., Dasari R.R., Feld M.S., Kuriabova T., Henle M.L., Levine A.J., Popescu G. (2010). Measurement of red blood cell mechanics during morphological changes. Proc. Natl. Acad. Sci. USA.

[227] Li J., Dao M., Lim C.T., Suresh S. (2015). Spectrin-level modeling of the cytoskeleton and optical tweezers stretching of the erythrocyte. Biophys. J..

[228] Discher D.E., Boal D.H., Boey S.K. (1998). Simulations of the erythrocyte cytoskeleton at large deformation. II. Micropipette aspiration. Biophys. J..

[229] Lim G.H.W., Wortis M., Mukhopadhyay R. (2008). Red Blood Cell Shapes and Shape Transformations: Newtonian Mechanics of a Composite Membrane: Sections 2.1–2.4. Soft Matter: Lipid Bilayers and Red Blood Cells.

[230] Khairy K.J., Foo J.J., Howard J. (2010). Shapes of red blood cells: Comparison of 3D confocal images with the bilayer-couple model. Cell. Mol. Bioeng..

[231] Albu R.M., Avram E., Stoica I., Ionid E.G., Popovici D., Ioan S. (2011). Surface properties and compatibility with blood of new quaternized polysulfones. J. Biomater. Nanobiotechnol..

[232] Reinhart W.H., Piety N.Z., Goede J.S., Shevkoplyas S.S. (2015). Effect of osmolality on erythrocyte rheology and perfusion of an artificial microvascular network. Microvasc. Res..

[233] Burns J.M., Yang X., Forouzan O., Sosa J.M., Shevkoplyas S.S. (2012). Artificial microvascular network: A new tool for measuring rheologic properties of stored red blood cells. Transfusion.

[234] Shevkoplyas S.S., Yoshida T., Gifforda S.C., Bitensky M.W. (2006). Direct measurement of the impact of impaired erythrocyte deformability on microvascular network perfusion in a microfluidic device. Lab Chip.

[235] Bleilevens C., Lölsberg J., Cinar A., Knoben M., Grottke O., Rossaint R., Wessling M. (2018). Microfluidic cell sorting: Towards improved biocompatibility of extracorporeal lung assist devices. Sci. Rep..

[236] Kang Y.J., Lee S.-J. (2018). In vitro and ex vivo measurement of the biophysical properties of blood using microfluidic platforms and animal models. Analyst.

[237] Suwanpayak N., Jalil M.A., Aziz M.S., Ismail F.D., Ali J., Yupapin P.P. (2011). Blood cleaner on-chip design for artificial human kidney manipulation. Int. J. Nanomed..

[238] Lange J.R., Metzner C., Richter S., Schneider W., Spermann M., Kolb T., Whyte G., Fabry B. (2017). Unbiased high-precision cell mechanical measurements with microconstrictions. Biophys. J..

[239] Antonova N., Riha P., Ivanov I. (2008). Time dependent variation of human blood conductivity as a method for an estimation of RBC aggregation. Clin. Hemorheol. Microcirc..

[240] Zhang W., Zhang Y.S., Bakht S.M., Aleman J., Shin S.R., Yue K., Sica M., Ribas J., Duchamp M., Ju J. (2016). Elastomeric free-form blood vessels for interconnecting organs on chip systems. Lab Chip.

[241] Ronco C. (2015). Hemodiafiltration: Technical and clinical issues. Blood Purif..

[242] Canaud B., Bosc I.Y., Cabrol L., Leray-Moragues H., Navino C., Verzetti G., Thomaseth K. (2000). Urea as a marker of adequacy in hemodialysis. Lesson from in vivo urea dynamics monitoring. Kidney Int. Suppl..

[243] Ahlmen J. (2004). Quality of life of the dialysis patient. Replacement of Renal Function by Dialysis.

[244] Pereira B.J.G., Cheung A.K., Lameire N., Mehta R.L. (2000). Complications of biocompatibility of hemodialysis membranes. Complications of Dialysis.

[245] Voinova M.V., Gorelik L.Y., Sokol E.I., Yurish S.Y. (2018). Sensors and bioelectronics in the kidney replacement therapy applications. Chapter 2. Advances in Biosensors: Reviews.

[246] Koda Y., Saito A., Kawanishi H., Yamashita A.C., Mineshima M. (2011). Clinical benefits of high-performance membrane dialyzers. High-Performance Membrane Dialyzers.

[247] Fernández E.A., Valtuille R., Balzarini M., Azar A.T. (2013). Artificial Neural Networks Applications in Dialysis. Modeling and Control of Dialysis Systems.

[248] Hoenich N.A., Ghezzi P.M., Ronco C., Hörl W.H., Koch K.M., Lindsay R.M., Ronco C., Winchester J.F. (2004). Hemodialyzers and related devices. Replacement of Renal Function by Dialysis.

[249] Humes H.D., Fissell W.H., Tiranathanagul K. (2006). The future of hemodialysis membranes. Kidney Int..

[250] Gambro Healthcare/Diaverum. https://www.baxter.com.

[251] Fresenius https://www.fresenius.com.

[252] Asahi Kasei https://www.asahi-kasei.co.jp/asahi/en/.

[253] Nephros https://www.nephros.com.

[254] Bouré T., Vanholder R. (2004). Which dialyser membrane to choose?. Nephrol. Dialys. Transplant..

[255] Barzin J., Feng C., Khulbe K.C., Matssura T., Madaeni S.S., Mirzadeh H. (2004). Characterization of polyethersulfone hemodialysis membrane by ultrafiltration and atomic force microscopy. J. Membr. Sci..

[256] Eloot S., De Wachter D., Vienken J., Pohlmeier R., Verdonck P. (2002). In vitro evaluation of the hydraulic permeability of polysulfone dialysers. Int. J. Artif. Organs.

[257] Kaysen G.A., Zhu F., Sarkar S., Heymsfield S.B., Wong J., Kaitwatcharachai C., Kuhlmann M.K., Levin N.W. (2005). Estimation of total-body and limb muscle mass in hemodialysis patients by using multifrequency bioimpedance spectroscopy. Am. J. Clin. Nutr..

[258] Sanchidrián S.G., Gómez P.J.L., Álvarez J.P.M., Herrero M.C.J., Cerviño I.C., Domínguez S.G. (2016). Reacción a membranas sintéticas en hemodiálisis. Nefrologia.

[259] Kim J.Y., Lee H.K., Kim S.C. (1999). Surface structure and phase separation of polysulfone membrane by atomic force microscopy. J. Membr. Sci..

[260] Álvarez-de Lara M.A., Martín-Malo A. (2014). Hypersensitivity reactions to synthetic haemodialysis membranes—An emerging issue?. Nefrologia.

[261] Šefer S., Vidović L. (2017). Allergic reactions to polysulfone dialysis membranes—An old problem taking a new dimension?. Acta Med. Croatica.

[262] Vienken J., Peinemann K.-V., Nunes S.P. (2008). Membranes in hemodialysis. Membrane Technology.

[263] Corbatón-Báguena M.-J., Álvarez-Blanco S., Vincent-Vela M.-C. (2015). Fouling mechanisms of ultrafiltration membranes fouled with whey model solutions. Desalination.

[264] Koivu V., Decain M., Geindreau C., Mattila K., Bloch J.F., Kataja M. (2011). Transport properties of heterogeneous materials. Combining computerised X-ray micro-tomography and direct numerical simulations. Int. J. Comput. Fluid Dyn..

[265] Matsuda T. (1989). Biological response at non-physiological interfaces and molecular design of biocompatible surfaces. Nephrol. Dial. Transplant..

[266] Chanard J., Lavaud S., Randoux C., Rieu P. (2003). New insights in dialysis membrane biocompatibility: Relevance of adsorption properties and heparin binding. Nephrol. Dial. Transplant..

[267] Namekawa K., Schreiber M.T., Aoyagi T., Ebara M. (2014). Fabrication of zeolite-polymer composite nanofibers for removal of uremic toxins from kidney failure patients. Biomater. Sci..

[268] Saito A., Foley H.C. (1991). Curvature and parametric sensitivity in models for adsorption in micropores. AIChE J..

[269] Qajar A., Daigle H., Prodanović M. (2016). The effects of pore geometry on adsorption equilibrium in shale formations and coal-beds: Lattice density functional theory study. Fuel.

[270] Fang A., Kroenlein K., Riccardi D., Smolyanitsky A. (2019). Highly mechanosensitive ion channels from graphene-embedded crown ethers. Nat. Mater..

[271] Gotch F.A., Sargent J.A. (1985). A mechanical analysis of the National Cooperative Dialysis Study (NCDS). Kidney Int..

[272] Gotch F.A. (1987). Kinetics of solute removal in hemodialysis. Adv. Exp. Med. Biol..

[273] Gotch F.A. (1975). Recommendations for quantification of dialysis therapy in research protocols. Kidney Int. Suppl..

[274] Sargent J.A. (1983). Control of dialysis by a single-pool urea model: The National Cooperative Dialysis Study. Kidney Int. Suppl..

[275] Flanigan M.J., Fangman J., Lim V.S. (1991). Quantitating hemodialysis: A comparison of three kinetic models [see comments]. Am. J. Kidney Dis..

[276] Popovich R.P., Hlavinka D.J., Bomar J.B., Moncrief J.W., Decherd J.F. (1975). The consequences of physiological resistance on metabolic removal from the patient-artificial kidney system. ASAIO.

[277] Daugirdas J.T., Depner T.A., Greene T., Silisteanu P. (2009). Solute-solver: A web-based tool for modeling urea kinetics for a broad range of hemodialysis schedules in multiple patients. Am. J. Kidney Dis..

[278] Daugirdas J.T., Schneditz D. (1995). Overestimation of hemodialysis dose depends on dialysis efficiency by regional blood flow but not by conventional two pool urea kinetic analysis. ASAIO.

[279] Daugirdas J.T., Smye S.W. (1997). Effect of a two compartment distribution on apparent urea distribution volume. Kidney Int..

[280] Daugirdas J.T., Blake G., Ing T.S. (2001). Handbook of Dialysis.

[281] Waniewski J. (1999). Mathematical models for peritoneal transport characteristics. Perit. Dial. Int..

[282] Waniewski J. (2006). Mathematical modeling of fluid and solute transport in hemodialysis and peritoneal dialysis. J. Membr. Sci..

[283] Waniewski J., Antosiewicz S., Baczynski D., Poleszczuk J., Pietribiasi M., Lindholm B., Wankowicz Z. (2016). Peritoneal fluid transport rather than peritoneal solute transport associates with dialysis vintage and age of peritoneal dialysis patients. Comput. Math. Methods Med..

[284] Waniewski J., Debowska M., Lindholm B. (2010). Can the diverse family of dialysis adequacy indices be understood as one integrated system?. Blood Purif..

[285] Waniewski J., Heimburger O., Werinski A., Lindholm B. (1996). Simple models for fluid transport during peritoneal dialysis. Int J. Artif. Organs..

[286] Waniewski J., Lucjanek P., Werynski A. (1994). Impact of ultrafiltration on back-diffusion in hemodialyzer. Artif. Organs..

[287] Waniewski J., Stachowska-Pietka J., Flessner M.F. (2009). Distributed modeling of osmotically driven fluid transport in peritoneal dialysis: Theoretical and computational investigations. Am. J. Physiol. Heart Circ. Physiol..

[288] Waniewski J., Werynski A., Heimburger O., Lindholm B. (1991). A comparative analysis of mass transport models in peritoneal dialysis. ASAIO Trans..

[289] Debowska M., Waniewski J., Lindholm B. (2005). Dialysis adequacy indices for peritoneal dialysis and hemodialysis. Adv. Perit. Dial..

[290] Debowska M., Lindholm B., Waniewski J., Carpi A., Donadio C., Tramonti G. (2011). Kinetic modeling and adequacy of dialysis. Progress in Hemodialysis—From Emergent Biotechnology to Clinical Practice.

[291] Velasco M., Azar A.T. (2013). Flow modeling of hollow fiber dialyzers. Modeling and Control of Dialysis Systems.

[292] Grzegorzewska A.E., Azar A.T., Roa L.M., Oliva J.S., Milán J.A., Palma A., Azar A.T. (2013). Single pool urea kinetic modeling. Modeling and Control of Dialysis Systems.

[293] Azar A.T., Yashiro M., Schneditz D., Roa L.M., Azar A.T. (2013). Double pool urea kinetic modeling. Modeling and Control of Dialysis Systems.

[294] Bruggeman D.A.G. (1935). Berechnung verschiedener physikalischer Konstanten von heterogenen Substanzen. I. Dielektrizitätskonstanten und Leitfähigkeiten der Mischkörper aus isotropen Substanzen. Annalen der Physik.

[295] Hanai T. (1960). Theory of the dielectric dispersion due to the interfacial polarization and its applications to emulsions. Kolloid Z..

[296] Hanai T., Koizumi N. (1975). Dielectric relaxation of W/O emulsions in particular reference to theories of interfacial polarization. Bull. Inst. Chem. Res. Kyoto Univ..

[297] Looyenga H. (1965). Dielectric constants of heterogeneous mixture. Physica.

[298] De La Rue R.E., Tobias C.W. (1959). On conductivity of dispersions. J. Electrochem. Soc..

[299] Fricke H. (1953). The Maxwell-Wagner dispersion in a suspension of ellipsoids. J. Phys. Chem..

[300] Varlet-Marie E., Brun J.F. (2011). Prediction of RBC aggregability and deformability by whole body bioimpedance measurements analyzed according to Hanai’s mixture conductivity theory. Clin. Hemorheol. Microcirc..

[301] Ogston A.G. (1958). The spaces in a uniform random suspension of fibres. Trans. Faraday Soc..

[302] Lorenzo A.D., Andreoli A., Matthie J., Withers P. (1997). Predicting body cell mass with bioimpedance by using theoretical methods: A technological review. J. Appl. Physiol..

[303] Matthie J.R. (2005). Second generation mixture theory equation for estimating intracellular water using bioimpedance spectroscopy. J. Appl. Physiol..

[304] Matthie J.R. (2008). Bioimpedance measurements of human body composition: Critical analysis and outlook. Expert Rev. Med. Devices..

[305] Jaffrin M.Y., Morel H. (2008). Body fluid volumes measurements by impedance: A review of bioimpedance spectroscopy (BIS) and bioimpedance analysis (BIA) methods. Med. Eng. Phys..

[306] Jaffrin M.Y., Fenech M., Moreno M.V., Kieffer R. (2006). Total body water measurement by a modification of the bioimpédance spectroscopy method. Med. Biol. Eng. Comput..

[307] Starling E.H. (1896). On the absorption of fluids from the connective tissue spaces. J. Physiol..

[308] Staverman A.J. (1951). The theory of measurement of osmotic pressure. Rac. Trav. Chim..

[309] Hu X., Adamson R.H., Liu B., Curry F.E., Weinbaum S. (2000). Starling forces that oppose filtration after tissue oncotic pressure is increased. Am. J. Physiol. Heart Circ. Physiol..

[310] Landau L.D., Lifshitz E.M. (1986). Theory of Elasticity. Course of Theoretical Physics.

